# Three-dimensional electron microscopy reconstruction and cysteine-mediated crosslinking provide a model of the type III secretion system needle tip complex

**DOI:** 10.1111/mmi.12843

**Published:** 2014-11-27

**Authors:** Martin Cheung, Da-Kang Shen, Fumiaki Makino, Takayuki Kato, A Dorothea Roehrich, Isabel Martinez-Argudo, Matthew L Walker, Isabel Murillo, Xia Liu, Maria Pain, James Brown, Gordon Frazer, Judith Mantell, Petros Mina, Thomas Todd, Richard B Sessions, Keiichi Namba, Ariel J Blocker

**Affiliations:** 1 Schools of Cellular & Molecular Medicine and Biochemistry, University of BristolBristol, BS8 1TD, UK; 2 Graduate School of Frontier Biosciences, University of OsakaOsaka, 565-0871, Japan; 3 MLW Electron Microscopy Consulting LauncestonCornwall, PL15 9BB, UK; 4 Wolfson Bioimaging Facility, School of Biochemistry, University of BristolBristol, BS8 1TD, UK; 5 Bristol Centre for Complexity Sciences, Predictive Sciences Helpdesk, University of BristolBristol, BS8 1TR, UK; 6 School of Biochemistry, University of BristolBristol, BS8 1TD, UK; 7 Riken Quantitative Biology CenterOsaka, 565-0871, Japan

## Abstract

Type III secretion systems are found in many Gram-negative bacteria. They are activated by contact with eukaryotic cells and inject virulence proteins inside them. Host cell detection requires a protein complex located at the tip of the device's external injection needle. The *Shigella* tip complex (TC) is composed of IpaD, a hydrophilic protein, and IpaB, a hydrophobic protein, which later forms part of the injection pore in the host membrane. Here we used labelling and crosslinking methods to show that TCs from a Δ*ipaB* strain contain five IpaD subunits while the TCs from wild-type can also contain one IpaB and four IpaD subunits. Electron microscopy followed by single particle and helical image analysis was used to reconstruct three-dimensional images of TCs at ∼20 Å resolution. Docking of an IpaD crystal structure, constrained by the crosslinks observed, reveals that TC organisation is different from that of all previously proposed models. Our findings suggest new mechanisms for TC assembly and function. The TC is the only site within these secretion systems targeted by disease-protecting antibodies. By suggesting how these act, our work will allow improvement of prophylactic and therapeutic strategies.

## Introduction

Gram-negative bacteria use type III secretion systems (T3SSs) as protein transport devices for injecting virulence effector proteins into eukaryotic cells during infection (Kosarewicz *et al*., 2012). T3SSs consist of a ‘needle complex’ (NC; Kubori *et al*., 1998) composed of a basal body commencing in the bacterial cytoplasm and spanning both prokaryotic membranes, into which a hollow extracellular needle is embedded that serves as secretion channel. The tip complex (TC; Mueller *et al*., 2005; 2008) tops the distal end of the needle. It is required for formation of a pore within the plasma membrane of host cells, which is necessary for effector translocation.

Our model system, *Shigella flexneri*, the agent of human bacillary dysentery, uses its T3SS to invade gut epithelial cells. As for many pathogens, its T3S apparatus is assembled when environmental conditions are appropriate for invasion, but secretion is blocked until physical contact with a host cell generates an activation signal (Schroeder and Hilbi, 2008). Despite increasing evidence that the needle and TC are involved in host cell sensing (Blocker *et al*., 2008; Roehrich *et al*., 2013), the nature of the activation signal and its mode of transmission remain unsolved mysteries, not least because an understanding of TC organisation has been difficult to obtain.

The TC was first visualised in two dimensions in *Yersinia* (Mueller *et al*., 2005). In *Shigella*, it is composed of two proteins, each essential to host cell sensing: IpaD (Espina *et al*., 2006; Sani *et al*., 2007; Veenendaal *et al*., 2007; Roehrich *et al*., 2010) and IpaB (Olive *et al*., 2007; Veenendaal *et al*., 2007; Shen *et al*., 2010). Protein translocation into host cells additionally requires IpaC, which is recruited to the TC upon activation (Epler *et al*., 2009; Veenendaal *et al*., 2007; Roehrich *et al*., 2010; Shen *et al*., 2010). These proteins are collectively called the translocators. IpaD is hydrophilic and required for tip recruitment of the hydrophobic proteins IpaB and IpaC, which later form the pore in host cell membranes. However, when a hydrophobic translocator is found within the *Yersinia* TC it is an IpaC, not an IpaB, homolog (Mueller *et al*., 2005; Harmon *et al*., 2013). Furthermore, there is disagreement as to when IpaB is added to the *Shigella* TC. Others suggested this occurs after exposure to the bile salt deoxycholate (DOC; Olive *et al*., 2007; Stensrud *et al*., 2008), whereas we do not require DOC treatment to find IpaB in TCs (Veenendaal *et al*., 2007).

The *Shigella* needle protein, MxiH, is a ∼ 9 kDa, α-helical hairpin (Fujii *et al*., 2012; Demers *et al*., 2013). It polymerises into the helical needle, containing ∼ 5.6 subunits/turn, using both of its α-helical termini (Cordes *et al*., 2003; Fujii *et al*., 2012). The subunit is oriented with its N-terminus on the outside of the polymer (Loquet *et al*., 2012; Demers *et al*., 2013). The initial atomic model of the needle derived from solid-state nuclear magnetic resonance (ssNMR) (Loquet *et al*., 2012; Demers *et al*., 2013) did not fit fully into our 7 Å density map of the needle obtained by electron cryomicroscopy (cryoEM) (Fujii *et al*., 2012; Abrusci *et al*., 2014). However, a more recent atomic model of the needle by combining ssNMR and cryoEM seems to show an improved fit (Demers *et al*., 2014). Single amino acid mutations in the needle protein lead to alterations in secretion regulation and TC composition (Kenjale *et al*., 2005; Torruellas *et al*., 2005; Veenendaal *et al*., 2007), indicating the needle and its tip are functionally coupled. Similar to the needle protein, the ∼37 kDa IpaD contains a central coiled coil and requires mostly its extreme C-terminus to bind the needle (Johnson *et al*., 2007; Veenendaal *et al*., 2007). In addition, it has two globular domains, one at the N-terminus, which acts as an intrabacterial self-chaperone, and the other linking the two helices of the coiled coil (Johnson *et al*., 2007). Point mutations in the upper part of the IpaD C-terminal helix render the T3SS unable to respond to its artificial inducer, the small amphipathic dye Congo red (CR) (Bahrani *et al*., 1997), or to sense host cells (Roehrich *et al*., 2013). Finally, the structure of ∼62 kDa IpaB remains mostly unknown: only one third was crystallised, as an ∼150 amino acid-long antiparallel coiled coil (Barta *et al*., 2012).

**Fig. 1:**
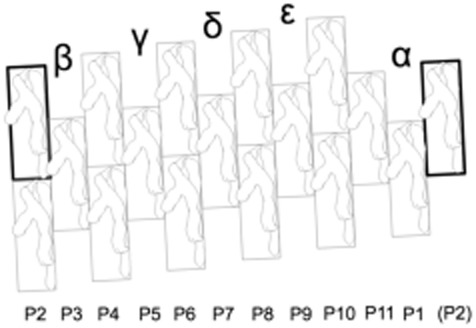
MxiH needle protofilament nomenclature assignments used in this study. 2D representation of the needle filament, with protofilaments labeled from P1-P11, with P1 corresponding to the lowest protofilament in the helical arrangement. Subunits boxed in bold line are at an identical location in the needle helix. Note that TC subunits will later be labelled from α → ε according to their proposed order of insertion (with α being the lowest and hence probably the first subunit inserted).

At the helical needle tip, the 11 MxiH protofilaments (in this study denoted P1-P11 from the lowest, most bacterial-proximal one, to the highest; Fig. 1) generate five new subunit-binding sites. Four out of the five potential protein insertion sites are equivalent but the lowest is structurally unique because it is bound by two non-continuously rising subunits. We therefore proposed that four IpaD molecules polymerise at the needle tip, while IpaB fills the unique site, again binding the needle via its extreme C-terminus (Johnson *et al*., 2007; Veenendaal *et al*., 2007; Blocker *et al*., 2008; Roehrich *et al*., 2010). This model puts the predicted central coiled coil and C-terminal globular domains of IpaB in a topologically equivalent position to those of IpaD atop the TC, optimally positioning its hydrophobic regions to interact with host cell membranes (Johnson *et al*., 2007; Roehrich *et al*., 2010; Shen *et al*., 2010).

However, the six recent structural studies examining this hypothesis are discordant (Dickenson *et al*., 2010; 2013[Bibr b17],[Bibr b18]; Wang *et al*., 2010; Barta *et al*., 2011; Chatterjee *et al*., 2011; Lunelli *et al*., 2011; Abrusci *et al*., 2014). Each of three crystallography and two NMR studies reveals a different interaction of IpaD or its *Salmonella* homolog SipD with DOC, with one of these also describing the crystal structure of a PrgI (*Salmonella* needle protein)-SipD fusion claimed to represent ‘open’, activated TCs. These differences probably arise from the helical needle-TC interface being an unsuitable crystallographic or solution NMR target. Furthermore, three-dimensional image reconstruction (3DR) by electron microscopy (EM) and single particle image analysis of the tips of *Shigella* needles sheared off bacteria lead to density maps where the helical features of the needle surface are not apparent and the proposed TC end displays rotational symmetry. An IpaD pentamer could only be docked at this location with major rearrangements of the C-terminal globular domains (Epler *et al*., 2012). This is probably because the TC is small, featureless and asymmetric (Veenendaal *et al*., 2007). We describe herein how these difficulties were surmounted, revealing the asymmetry of the TC and providing novel insights into how it assembles and functions. This new knowledge has important therapeutic implications since the TC is the only part of T3SSs that has been successfully immunologically targeted for use in humans so far (Sato and Frank, 2011; Milla *et al*., 2013).

## Results

### Cysteine crosslinking establishes the relative orientation of IpaD subunits within the TC

**Fig. 2:**
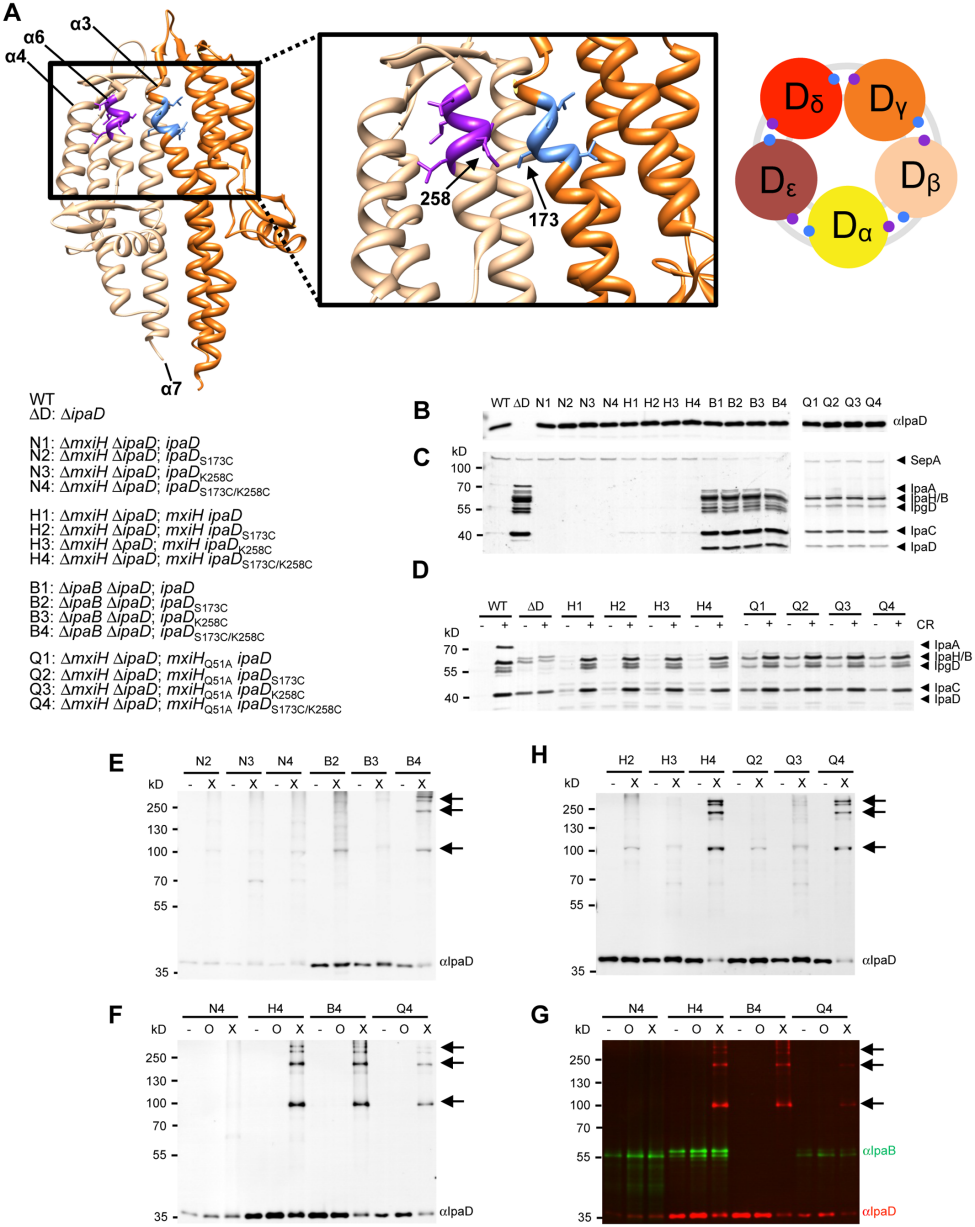
Analysis of the organisation of IpaD subunits within the TC by chemical crosslinking.A. Model of IpaD dimer (*left*; PDB ID 2j0n) and close up view (*middle*) displaying the regions V170 – L174 of helix α3 (blue) of one subunit and L257 – D261 of helix α6 (purple) of the second subunit used for cysteine mutagenesis. *Right*, schematic top view of TC atop the needle, identifying the predicted relative location of the mutations made. IpaD subunits are labelled from lighter to darker colours (i.e. yellow, pale orange, dark orange, red and maroon) and using alphabetically ordered Greek letters according their increasing height in the TC. Strains used for crosslinking were verified by analysing the expression level of IpaD via Western blot (B), the secretion of Ipa proteins from the supernatant (C) and after induction with Congo red (CR) (D) via silver staining. Crosslinking analysis of IpaD on the tip of the needles within crudely purified NCs (E–H) in the absence (−) or presence of crosslinker BM(PEG)_2_ (X) or oxidiser Na_2_S_4_O_6_ (O) via Western blot using antibodies against IpaD and IpaB. (G) Same Western blot as in F, exposed to show the anti-IpaB signal (green) together with the anti-IpaD one (red). All data shown are representative of at least 2 independent experiments. The strains used for these assays, and how they are symbolised in panels B–G, are listed to the left of panels B–D.

To establish the orientation of the IpaD subunits atop needles, we analysed the interaction of cysteine point mutations inserted within them. Single cysteine mutations were generated in regions V170 – L174 of IpaD helix α3 and L257 – D261 of helix α6. Subsequently, combinatorial double cysteine mutations were generated in these regions. The design of these mutations was based on a dimer, observed in one of the IpaD crystal forms. This dimer was hypothesised, without experimental assessment until now, to represent relative subunit orientations in the TC (Johnson *et al*., 2007; Blocker *et al*., 2008). In this dimer, the regions of helices α3 and α6 specified above would appose each other tightly (Fig. 2A) and should therefore interact chemically.

IpaD contains an intrinsic cysteine within its C-terminus (position 322). To prevent ambiguity, this was mutated to serine, without phenotypic effect when expressed in an Δ*ipaD* background (Picking *et al*., 2005). Hence, the background Δ*ipaD*/*ipaD*_C322S_ is denoted simply *ipaD* in Supporting Table S1, Fig. 2 and Supporting Fig. S1. Most single and double *ipaD* mutants were phenotypically normal in standard assays of *Shigella* T3SS functionality (protein expression, low level Ipa protein secretion and contact hemolysis, which measures translocon insertion) except for those containing L171C, which displayed slightly reduced hemolytic efficiencies (Fig. S1A and B).

We then sought to form disulphide bonds between adjacent subunits atop needles from these mutants using the *oxidiser* sodium tetrathionate to form disulphide bonds or to chemically crosslink them using a non-cell permeable, non-reversible, cysteine *crosslinker* with an 8–15 Å spacer arm, 1,8-bis(maleimido)diethylene glycol (BM(PEG)_2_) (Green *et al*., 2001). To ease the screening of these 35 strains, we developed a protocol to crosslink TCs using crudely purified long needles by overexpression of *mxiH*, denoted by a * in Supporting Tables S1 and S3, as an extension of a method we had used to examine TCs in the past (Veenendaal *et al*., 2007). The state of IpaD was monitored by Western blot. This procedure generated an IpaD signal only when *mxiH* was overexpressed, indicating the method detected primarily needle- and hence TC-associated IpaD (Fig. S1C). In the presence of the crosslinker, for certain double mutants, a main band at approximately 100 kDa was seen. This was assumed to be an IpaD dimer migrating abnormally slowly since it was never seen in any of the single mutants (Supporting Table S3). On occasion, a band migrating at approximately 170 kDa and probably corresponding to IpaD oligomers was noted (Supporting Table S3). However, no higher molecular weight bands were observed in the presence of oxidiser. Paired mutations S170C/K258C, S172C/K258C, S172C/D261C, S173C/K258C, S173C/S259C and L174C/D261C generated strong bands corresponding to crosslinked product (Supporting Table S3). Given the length of the crosslinker arm and the diameter of an IpaD molecule, these pairs can only come from immediately adjacent TC subunits. Taken together, these data confirm that at least some of the subunits are arranged with α3 and α6 facing each other. However, they also indicate that, unlike in the earlier proposal (Johnson *et al*., 2007; Blocker *et al*., 2008), these are not close enough to allow disulphide bond formation (6–7 Å). Rather, the linked sulphur atoms must lie between 8 and 15 Å of each other.

From then on, we focused on analysis of the double mutant *ipaD*S173C/K258C, since it gave the strongest signals (as predicted by the initial model in Fig. 2A), in parallel with single mutants *ipaD*S173C and *ipaD*K258C. To probe the organisation of IpaD subunits within TCs in different mutant backgrounds, we constructed the strains listed in Fig. 2 (Supporting Table S1). We verified that these strains expressed identical levels of IpaD (Fig. 2B) and displayed the secretion phenotypes expected of them: Δ*mxiH* (symbolised by N, for Null) background, absence of even low level Ipa protein secretion (Fig. 2C); wild-type (H, for MxiH wild-type) background, low level Ipa protein secretion and inducibility (Fig. 2C and D); Δ*ipaB* background (B, for Δ*ipaB*), fast constitutive secretion (Fig. 2C, i.e. to levels similar to that of Δ*ipaD* and much higher than that of wild-type) and uninducibility, both defined by Veenendaal *et al*. (2007). To enhance the signals obtained, we also modified the experimental procedure to use crudely purified NCs and new anti-IpaD antibodies. This led to the reliable detection of four bands from *ipaD*S173C/K258C within an otherwise wild-type background treated with BM(PEG_2_) (X), but not with the oxidiser sodium tetrathionate (O): one at 100 kDa, one at 170 kDa, and two above 250 kDa (Fig. 2F and H, lanes H4X). Again, the size of these bands did not precisely correspond to those expected for multimers of IpaD. Yet it is not uncommon for artificially crosslinked proteins to migrate at abnormal molecular weights in sodium dodecyl sulfate polyacrylamide gel electrophoresis (SDS-PAGE). Indeed, each band must contain IpaD because it is recognised by a polyclonal antiserum affinity purified on recombinant IpaD and tested for mono-specificity. Furthermore, in all lanes within Fig. 2E, H and F where crosslinking occurred the amount of monomeric IpaD is greatly reduced, suggesting it is a major component of the new bands appearing after crosslinking. Finally, IpaD is very unlikely to be crosslinking to anything else but itself because: (i) MxiH contains no cysteine, (ii) IpaB, which carries a single cysteine within its central domain, was not observed to co-migrate with these bands (Fig. 2F and G, lane H4X) and (iii) the bands are only seen in specific double mutants, not in any of the single ones (Fig. 2E, F and H; Supporting Table S3). These bands therefore correspond to heterocrosslinks allowing formation of dimers, trimers, tetramers and pentamers of IpaD. Indeed, very similar results were obtained when these mutations were moved into a Δ*ipaB* background (Fig. 2E and F, lanes B4X). This suggests a significant proportion of wild-type TCs contain five IpaD subunits.

### TCs can exist as either homo- or heteromeric assemblies

The ability to crosslink five IpaD subunits within TCs supports previous reports that the *Shigella* TC is homopentameric (Espina *et al*., 2006; Epler *et al*., 2012). However, IpaB and IpaD are in a 1:5 to 1:10 molar ratio in isolated needles and antibodies to IpaB can crosslink TCs in NCs (Veenendaal *et al*., 2007). Moreover, cumulative evidence indicates IpaB is a key host cell-sensing component of TCs (Veenendaal *et al*., 2007; Roehrich *et al*., 2010; Shen *et al*., 2010).

To further investigate subunit composition and stoichiometry within the TC, we labelled IpaD and IpaB individually with avidin by inserting a 15 amino acid residue biotinylation signal into the N-terminus of each subunit between their T3SS secretion signal and chaperone-binding domain. The growth conditions of strains were optimised for maximal biotinylation and these were validated for TC functionality (using inducibility and epithelial cell invasion assays; Supporting Figs S2A–D and S3A and B). Purified NCs with avidin-labelled TCs were then prepared and validated as shown in Supporting Fig. S3C and D. Finally, the bound avidin was visualised by negative-stain EM. Despite the labelling efficiency of IpaD_avitag and IpaB_avitag being nearly 100% and 80%, respectively, when assayed biochemically (as described in the Supporting Results, under Optimisation of avidin binding, and shown in Fig. S3C and D), only 25% of *ipaD_avi_* (Fig. S4A–C and Supporting Information) and 2.5% of *ipaB_avi_* needle tips (Fig. S4C and D) were observed as labelled with at least one avidin. This implies in particular that most *ipaB_avi_* TCs did not carry an IpaB subunit. This discrepancy may be explained by the fact that neither protein is secreted at wild-type levels (Fig. S2A and B), perhaps due to partial disruption of their secretion signals/chaperone binding regions (Lokareddy *et al*., 2010). In addition, avidin-binding might destabilise Ipa subunits and/or their interaction with the needle tip. Indeed, in both data sets, the positions of the bound avidin molecules varied greatly in the y-axis (Fig. S4A and D), suggesting flexibility and/or partial unfolding in the N-termini of the proteins.

This variation precluded use of standard image classification procedures. For TCs from *ipaD*_avi_, bound avidin was visualised by reconstituting each image using eigenvectors to remove noise from the raw data and allow direct avidin visualisation (Supporting Information and Fig. S4B). The data set was then analysed manually to determine the number of particles with different numbers of bound avidin molecules. The maximum number of bound avidin molecules seen was 4 (Fig. S4B and C). Furthermore, the frequencies of 1, 2, 3 or 4 avidin molecules observed per TC were in excellent agreement with frequencies predicted if each avidin was binding independently to an IpaD molecule and the experiment thus modelled as a Poisson process (Supporting Information and Fig. S4C). Since the model allows for an infinitesimal, albeit non-zero, probability of detecting a 5th site, the data cannot be used to rule out the previously proposed presence of five IpaDs (Espina *et al*., 2006; Epler *et al*., 2012). However, we also visualised single avidin molecules bound to TCs when IpaB was labelled (Fig. S4D). These findings are supported by detection of IpaB labelling on the surface of wild-type, intact bacteria using flow cytometry (FACS), which is dependent not only on the presence of needles (Fig. S4E) but also on that of IpaD (not shown), as previously reported (Veenendaal *et al*., 2007; Shen *et al*., 2010). Taken together, these data confirm a subunit stoichiometry of four IpaDs and one IpaB in at least a subset of the WT TCs. As a wild-type IpaB is required for secretion regulation, inducibility and host cell sensing (Veenendaal *et al*., 2007; Roehrich *et al*., 2010; Shen *et al*., 2010), the 4 IpaD:1 IpaB TC subpopulation must represent the functionally relevant one.

### Organisation of TCs within needle mutants displaying altered secretion regulation

FACS was also used to assess the composition of TCs from strains carrying mutations in *mxiH* and displaying altered secretion regulation: *mxiH*_Q51A_ (symbolised by Q, for Q51A; Fig. 2C and D) or *mxiH*_P44A_, slow constitutive secretion (i.e. at levels higher than wild-type or H but lower than Δ*ipaB* or Δ*ipaD*; Fig. 1C; Veenendaal *et al*., 2007) with retention of inducibility and *mxiH*_P44A + Q51A_, slow constitutive secretion and uninducibility (not shown; Veenendaal *et al*., 2007). In comparison to WT or their wild-type background equivalent (*mxiG*^−/+^; see Supporting Table S1), they mostly showed reduced MxiH and hence proportionately diminished IpaD and IpaB labelling (Fig. S4E). This suggested that needle polymerisation defects (Cordes *et al*., 2005; Veenendaal *et al*., 2007) led to smaller numbers of complete TCs but that those completed were of similar composition to that of wild-type. This was further confirmed by Western blot, which indicated that IpaD and IpaB, but not IpaC (unlike previously reported in isolated needles; Veenendaal *et al*., 2007), were present in the NCs isolated from these strains (not shown). The absence of hydrophobic IpaC in NCs from these strains may be due to the fact that detergents are used to prepare NCs but not needles.

We next investigated the relative orientation of IpaD subunits in NCs in a Δ*ipaD* Δ*mxiH* background where *mxiH* mutants Q51A and P44A + Q51A could be co-expressed with mutant IpaDs. For the double cysteine mutant *ipaD*S173C/K258C (but not the single cysteine mutants) four distinct bands were detected in an *mxiH*_Q51A_ background (Fig. 2H and F, lanes Q4X). The observed bands were of a similar molecular weight to those observed from wild-type and Δ*ipaB*, suggesting that the TCs from wild-type, Δ*ipaB* and *mxiH*_Q51A_ mutant backgrounds have similar quaternary structures. We did not detect any higher molecular weight bands in an *mxiH*_P44A+Q51A_ mutant background (not shown), probably due to the reduced number of complete TCs in this strain (Fig. S4E). This made it impossible for us to ascertain TC organisation in this background. We then attempted to crosslink subunits in TCs activated using the artificial inducer CR. However, we did not see any change in the crosslinking pattern of wild-type upon CR addition, suggesting any changes it induces are too small to be detected with such a flexible crosslinker, are reversible, occur in only a minority of TCs or do not involve IpaD subunits (Supporting Fig. S1D). Taken together with our results in the *mxiH*_Q51A_ background, these data make large-scale changes in the relative organisation of IpaD subunits during TC activation seem unlikely.

### Three-dimensional reconstruction of Δ*ipaB* and wild-type TCs

To obtain further information about subunit organisation in the TC we then sought to visualise it in three dimensions. For this we used EM and single particle image analysis. Since the reconstruction challenges faced were similar to those encountered during the image analysis of the filament–cap complex of the bacterial flagellum (Yonekura *et al*., 2000), we used a similar methodology (see also Supporting Information and Fig. S5). TCs atop a needle portion were selected manually from negative-stain electron micrographs (Supporting Fig. S6A) of purified NCs (Supporting Table S4), positioning the needle tip at the same location within each image. A bespoke program was developed to determine relative particle positions in the x and y-axes simultaneously and optimise their alignment accordingly. Eigen-decomposition of the aligned images by multivariate statistical analysis revealed helical information in their needle portion (Supporting Fig. S6B), which was then used to classify the particles. Strong helical patterns and TC information were seen in most class averages (Supporting Fig. S6C). The helical pattern was used to determine the azimuthal orientation of each class average by comparison to projections of a 16 Å map of the needle derived from negative-stain micrographs (Cordes *et al*., 2003). An initial density map was generated by back projecting the aligned class averages. The helical parameters of the needle portion of this reconstruction were determined, and a new reference was generated, with the appropriate helical symmetry enforced only in the needle portion of the map, which was subsequently used for a new round of projection matching. This step was iterated five times, with the new reconstruction after each round used to establish a new reference map.

**Fig. 3:**
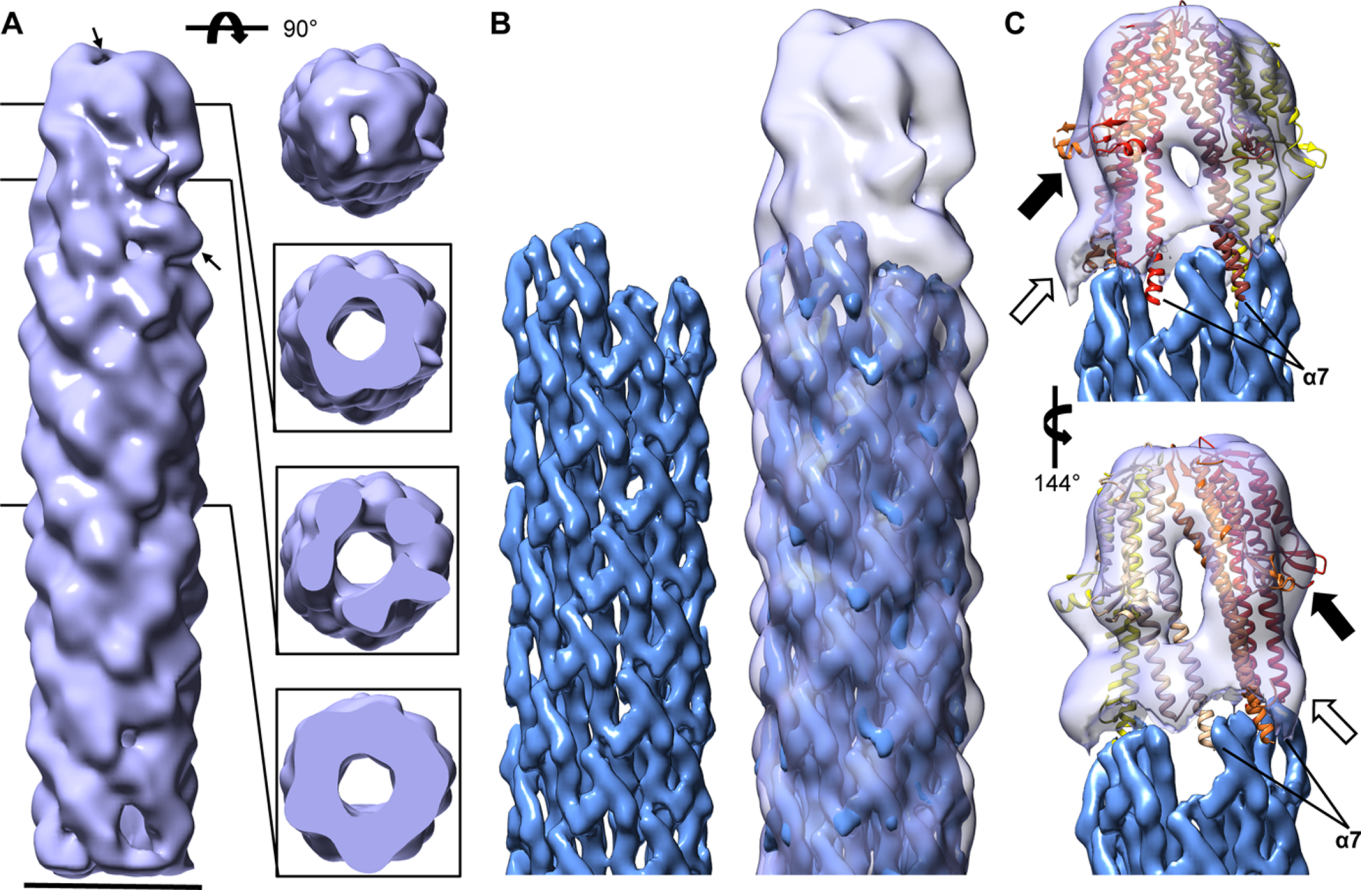
Three-dimensional reconstruction of the TC and needle from a Δ*ipaB* strain.A. 25 Å resolution electron density map of the TC and needle from Δ*ipaB* reconstructed using negative-stain EM, displaying view from the side (*left*) and top (*right*, *top*). Slices through the TC, TC/needle junction and needle portions are displayed of the *lower right* panels. *Thin arrows* indicate presumed location of IpaDαB. Electron density map of needle reconstructed using cryoEM (*left*; EMD-5352; Fujii *et al*., 2012) docked into the proposed needle portion of our TC-needle reconstruction (*right*).C. Isolated density of the TC following subtraction of the density corresponding to the needle. Five copies of a partial IpaD crystal structure (derived from PDB ID 2j0o (Johnson *et al*., 2007), molecule A as outlined in the main text) were docked into the individual subunit densities, their location colour coded as in Fig. 1A (*right*), i.e. the lightest subunit corresponding to the lowest position atop the needle helix. *Black arrows* indicate upper bulges assumed to correspond to the C-terminal globular domains of IpaD, while *white arrows* indicate lower bulges assumed to correspond to IpaD N-terminal domains (not found in the modified 2j0o structure). The new map is displayed throughout at a contour level of 0.0876 in Chimera. Scale bar, 70 Å.

To avoid complications arising from analysis of a heterogeneous population of TCs, we first reconstructed the 3D image of the TC from the Δ*ipaB* strain, which is only composed of IpaD subunits. The 25 Å-resolution density map of the Δ*ipaB* TC reveals an annular structure, of slightly larger diameter (∼80 Å) than that of the needle, where the first and second top view slices (from top) suggest five distinct subunits (Fig. 3A), all of similar size. Each subunit is tilted slightly to the left with respect to the needle axis (Fig. 3A, *left*), and they each differ somewhat in shape (Fig. 3A, *right*, top view and two inserts below), creating an asymmetric TC. However, the needle portion displays helical symmetry, with some resolution of the MxiH subunits (Fig. 3A, *left* and *right* bottom insert). The needle channel is well defined, extending through the length of the map. Indeed, top view analysis reveals a pore in the TC (Fig. 3A, *right*).

We used the helical pattern seen in the needle portion of the map to dock the 3DR of the needle (Fig. 3B, *right*) obtained by cryoEM (Fujii *et al*., 2012) (Fig. 3B, *left*). Subtraction of the needle density from the map allowed us to delineate the portion corresponding to the TC, which is approximately 110 Å high from bottom of the lowest subunit to the top of the highest one (Fig. 3C). We then docked into this portion five copies of a crystal structure of IpaD that lacks its first 38 and last 10 amino acids (Johnson *et al*., 2007), from which amino acids up to 125 were further removed (i.e. an IpaD fragment containing aa 126 to 322). The IpaD N-terminal domain is not required for it to bind to needles (Picking *et al*., 2005; Johnson *et al*., 2007; Veenendaal *et al*., 2007). Moreover, it acts as a self-chaperone and probably changes its location, and hence conformation, relative to the rest of the crystallised monomer when the protein assembles in the TC (Johnson *et al*., 2007). This makes the docking of that region treacherous, although the N-terminus was shown by immunolabelling (Veenendaal *et al*., 2007) and our avidin-labelling experiments to lie close to the needle portion on the TC surface, where five bulges are seen in lower part of the TC portion of the map (Fig. 3C, white arrows). Finally residues 323–332 were hand-built into an α-helix at the extreme C-terminus, as this region is strongly predicted to participate in IpaD's coiled coil and was not resolved in the crystal structure. Docking of these molecules showed that there was excellent correlation between the location of the globular C-terminal domain of IpaD and the five bulges seen on the surface of the upper portion of the TC map (Fig. 3C, black arrows). The C-terminus of helix α7 also fits well within the troughs created between each pair of contiguous MxiH molecules within the 1-start helix of the needle. Furthermore, there is adequate space to fit the remainder of the N-terminus of the protein within the unfilled lower bulges of the TC. Therefore, IpaD molecules are of an appropriate height to fit within our 3DR of the TC, where they can be docked without major rearrangement of their C-terminal domains.

**Fig. 4:**
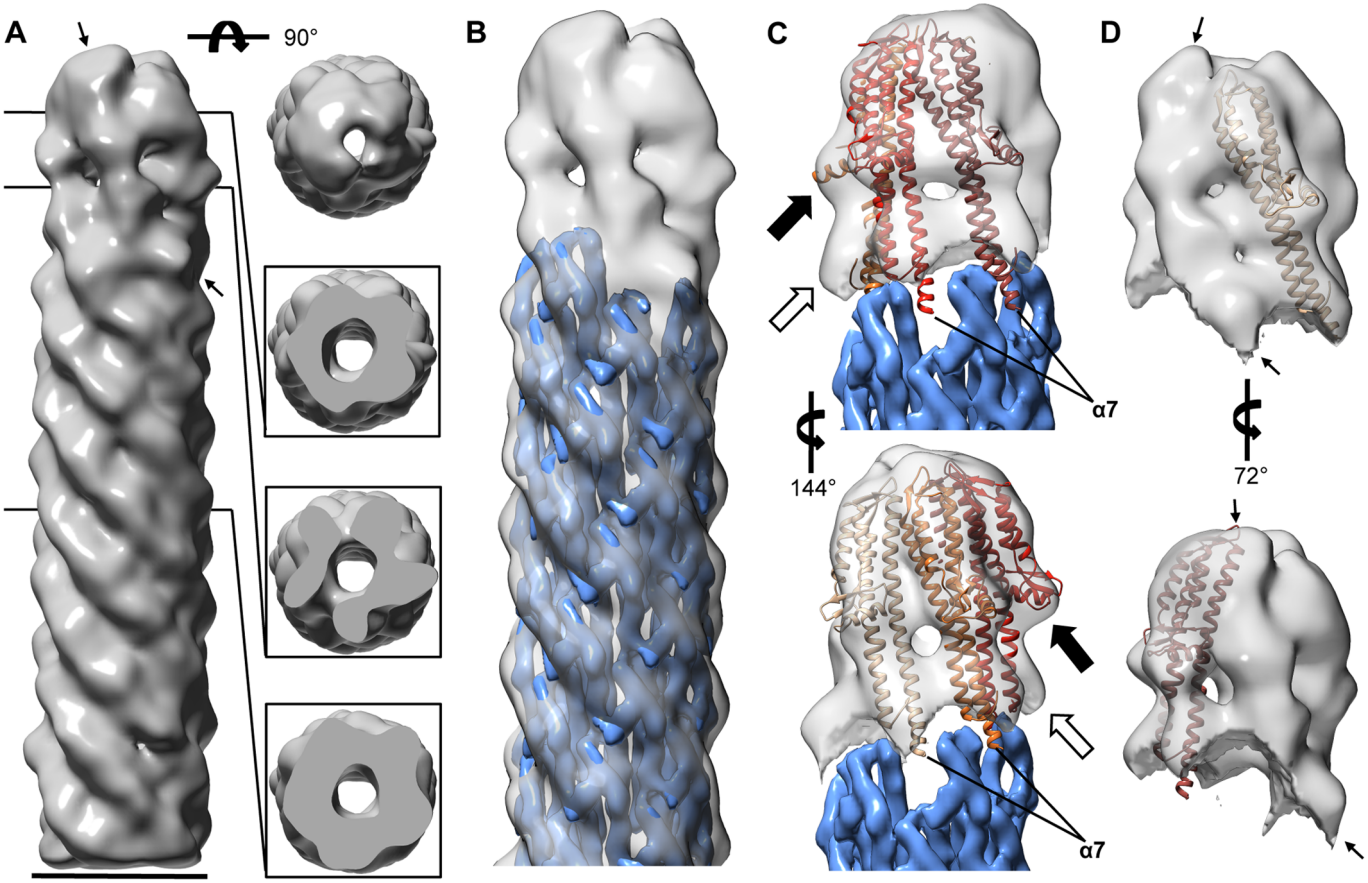
Three-dimensional reconstruction of the TC and needle from the wild-type strain.A. 24 Å resolution electron density map of the TC and needle from wild-type *Shigella* reconstructed using negative-stain EM, displaying view from the side (*left*) and top (*right*, *top*). Slices through the TC, TC/needle junction and needle portions are displayed of the *lower right* panels. *Thin arrows* indicate presumed location of IpaB.B. Electron density map of needle reconstructed using cryoEM (Fujii *et al*., 2012; EMD-5352) docked into the proposed needle portion of our TC-needle reconstruction with protofilament P1, containing the lowest MxiH subunit atop the needle helix below the highest and largest subunit in the TC.C. Isolated density of the TC following subtraction of the density corresponding to the needle. Four copies of a partial IpaD crystal structure (derived from PDB ID 2j0o (Johnson *et al*., 2007), molecule A as outlined in the main text) were docked into the individual subunit densities, their location colour coded as in Fig. 1A (*right*), i.e. the lightest subunit corresponding to position β. *Black arrows* indicate upper bulges assumed to correspond to the C-terminal globular domains of IpaD, while *white arrows* indicate lower bulges assumed to correspond to IpaD N-terminal domains (not found in the modified 2j0o structure).D. Isolated view of the wild-type TC with either IpaD_β_ (*top*) or IpaD_ε_ (*bottom*) docked in, to show larger size of subunit in position α. The new map is displayed throughout at a contour level of 0.0688 in Chimera. Scale bar, 70 Å. *Thin arrows* indicate presumed location of IpaB.

As wild-type TCs can exist as either homo- or heteropentamers and in order to obtain a map of the functionally relevant heteromeric tip, we used the TC map from the Δ*ipaB* strain to remove images that contained IpaD homopentameric tips from the wild-type data set. This was performed by first generating projections from the Δ*ipaB* map every 1° around the axis of the needle. The individual wild-type images were then aligned to these projections and sorted based on their degree of cross-correlation. The data set was split into two equally sized subsets according to the degree of cross-correlation and each set reconstructed independently. As predicted, the data set showing the higher cross-correlation generated a 3DR that looked similar to that of the TC from the Δ*ipaB* strain (not shown). Therefore, the one from the data set showing the lower cross-correlation should comprise primarily heteropentameric TCs. This map resolved to 24 Å resolution and also displayed a pentameric tip structure and clear helical pattern in the needle portion (Fig. 4A, *left*). In addition, cross-sections through the map showed similar features to corresponding sections of the Δ*ipaB* map (Fig. 4A, *right*). However, the highest subunit within the wild-type map is morphologically distinct from the others and from the equivalent one in the Δ*ipaB* map (Figs 3A and 4A, *left,* thin arrows). As we have shown that IpaB (62 kDa) is found within wild-type TCs and requires IpaD (37 kDa) to localise there (Veenendaal *et al*., 2007), we assumed that this larger subunit corresponded to IpaB. We therefore docked the cryoEM map of the needle with the lowest MxiH at the needle tip, from protofilament P1 (Supporting Fig. S7A), lying right below this subunit (Fig. 4B). After subtraction of the needle density, four IpaD subunits can be docked (Fig. 4C) into positions β to ε (Fig. 2A, *right* and Fig. S7A) of the TC, showing that the volume occupied by the subunit in position α is larger (Fig. 4D, thin arrows). Individual image classes used to generate both wild-type and Δ*ipaB* TC maps match specific reprojections of each map well, supporting the appropriateness of the reconstruction procedure (Fig. S7B and C).

### Refinement of the *mxiH*_Q51A_ TC map allows better visualisation of subunit composition and arrangement

We then performed EM image analysis of TCs from the aforementioned *mxiH* mutant strains, deemed to have TCs locked in different activation states (Kenjale *et al*., 2005; Veenendaal *et al*., 2007). For these reconstructions we were careful to pick TC particles only from NCs with needles of wild-type length, to ensure they also carried mature tips. This was confirmed by the fact that above 95% of the classes obtained had clear TCs visible (not shown). However, the data sets collected were too small to allow subtraction of homopentameric TC images without loss of reconstruction quality. For all mutants, the resulting density maps (Supporting Fig. S8A) showed similar resolutions (21–24 Å; Supporting Fig. S8B) with good helical patterns in the needle and clear annular, hollow TC structures atop the needles, again validating the reconstruction technique. Yet no significant differences were seen between the different mutant TCs at this resolution.

**Fig. 5:**
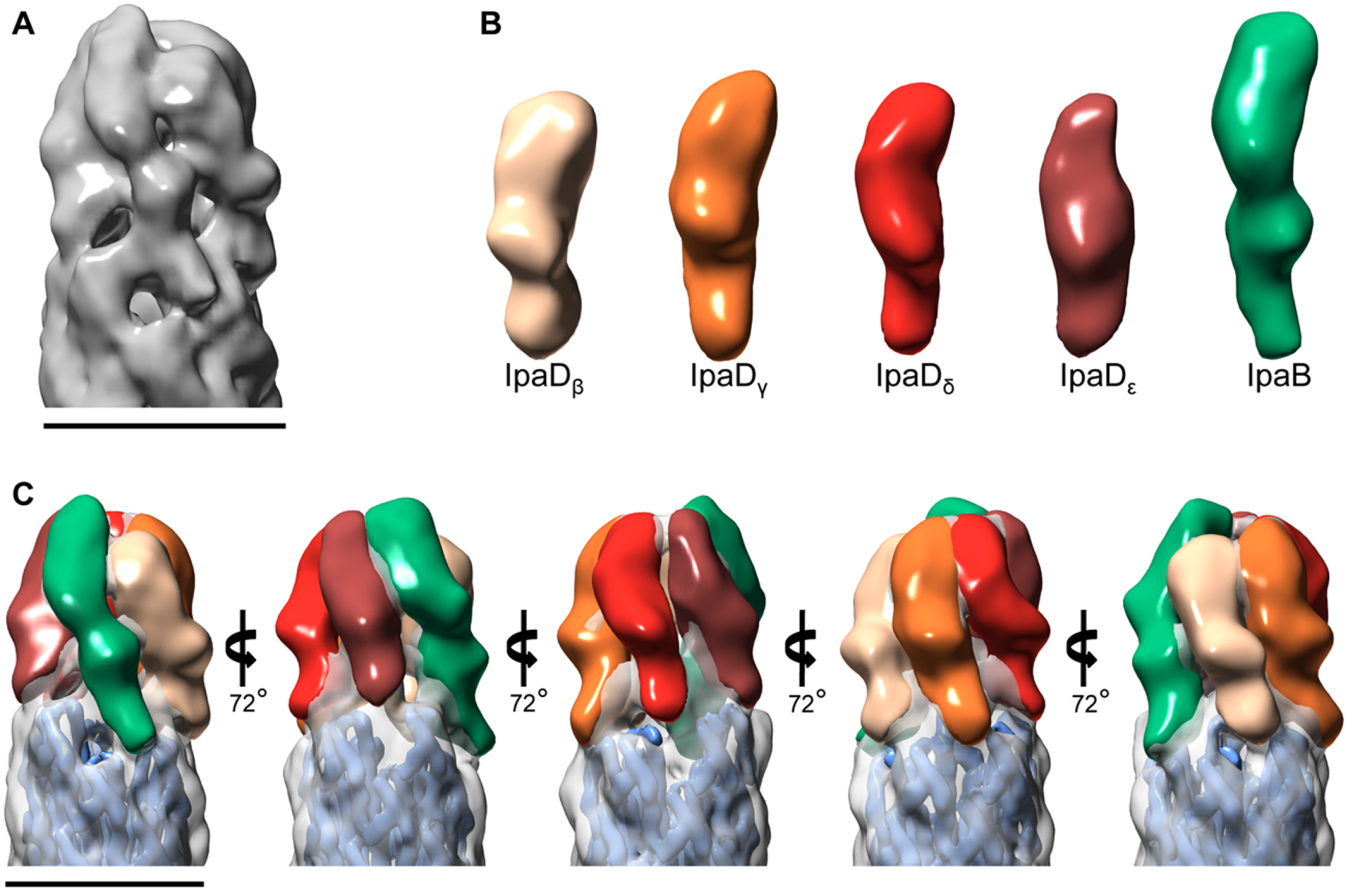
Refinement of the *mxiH_Q51A_* electron density map.A. The refined reconstruction shows five clear subunits, with one larger subunit that adopts the lowest position atop the needle but also extends furthest from the needle.B. The density of each individual subunit, separated from the rest of the map. Side-by-side comparison shows the difference in size between the larger subunit (green) and the four other subunits. The larger subunit is assumed to be IpaB, with four smaller IpaD subunits.C. Colour overlay of individual subunits on top of the 3D map shows the arrangement of TC subunits. Map displayed at a contour level of 0.0300 in Chimera. Scale bars, 70 Å; one in (A) also applies to (B).

Finally, we tried to improve the resolution of our reconstructions by refining the density maps. Using groups of single particles (rather than class averages), we realigned each particle image in the x and y-axes using another bespoke program before matching them with projections of only the TC portion of the density maps produced using class averages, to determine their azimuthal orientation. This worked best for the *mxiH*_Q51A_ data set, which was probably the best data set since its initial map resolved to 21 Å, allowing for improved resolution (19 Å; Supporting Fig. S8B) and hence better subunit definition (Fig. 5A). When separated from the TC, each smaller subunit appeared morphologically distinct, hinting at conformational differences between them. In addition, at position α, a larger subunit was unambiguously visualised as morphologically distinct from all others (Fig. 5B and C). Therefore, IpaB is bound to the lowest binding position atop the needle and also protrudes furthest from the TC top.

## Discussion

### IpaB is present in functional TCs

*Shigella* TCs are only functional for host cell sensing when they contain IpaB (Veenendaal *et al*., 2007; Roehrich *et al*., 2010; Shen *et al*., 2010). But, our avidin labelling and crosslinking data together suggest that a significant proportion of TCs examined here do not contain IpaB. As we did not see TCs with just 4 IpaD subunits, it is unlikely that IpaB is lost from TCs during the isolation procedure. This may hence instead reflect varying degrees of completeness and/or assembly stages. Alternatively, the lack of IpaB in some TCs could be an indication of the instability of this hydrophobic protein atop needles. In addition, when it is lost, it might be stochastically more likely to be replaced by an IpaD than by a new IpaB, at least initially. Whatever the reason may be, the heterogeneity identified here can explain why others have struggled to detect it at this location (Espina *et al*., 2006). This may also explain the low level T3SS-mediated Ipa protein secretion or ‘leakage’ observed in cultures of wild-type *Shigella*: IpaB might be regularly lost from the needle tip and, whilst it is absent or replaced by a secreted IpaD, the system is constitutive secretion mode (Menard *et al*., 1994).

We cannot estimate the percentage of TCs in our reconstructions that contain IpaB. However, we see a larger subunit within the wild-type after removal of the 50% of images correlating better with homopentameric IpaD TCs than the rest and in the refined *mxiH*_Q51A_ reconstructions. This suggests that the percentage of heteromeric tips is substantially more than the few percent indicated by the avidin labelling. In further support of the larger density corresponding to IpaB, the entire length of the recently published crystal structure of the IpaB antiparallel coiled coil (Barta *et al*., 2012) fits neatly into the larger subunit density (not shown). However, since this crystal structure is only a fragment of the entire IpaB structure, we cannot presently dock it accurately in our maps.

### Mode of TC assembly

Five subunits are seen in the Δ*ipaB* reconstruction (Fig. 3) whereas we expected to find only four copies of IpaD atop needles of this mutant. This is unlikely to be an artefact of the reconstruction procedure as we could match projections of the reconstruction with the contributing class averages (Supporting Fig. S7C). This suggests that the binding site for IpaB also permits binding of IpaD. Hence, during TC assembly, the initial IpaD -defined as IpaD_α_- may first bind to P1 (Supporting Fig. S7A), following the helical rise of the needle and its own mode of assembly, followed by sequential binding of IpaD_β_ to IpaD_ε_ to protofilaments P3, 5, 7 and 9, and finally displacement of IpaD_α_ by IpaB. This model is consistent with the finding that IpaB requires IpaD to bind needle tips (Veenendaal *et al*., 2007) and can explain why TCs exist as hetero- and homopentamers.

### The TC is open and flat

A striking observation for all our 3DRs is the presence of a channel and pore through the entire TC. Although the pore diameter is smaller (∼10 Å) than that of the needle channel (∼15 Å; Fujii *et al*., 2012), its occurrence is incongruent with the proposed ‘closed/open’ model of TC function (Veenendaal *et al*., 2007; Blocker *et al*., 2008; Lunelli *et al*., 2011) and might explain how low level secretion occurs prior to activation. It also supports the notion that T3SSs need to be additionally gated at their base (Lee *et al*., 2010; Martinez-Argudo and Blocker, 2010).

**Fig. 6:**
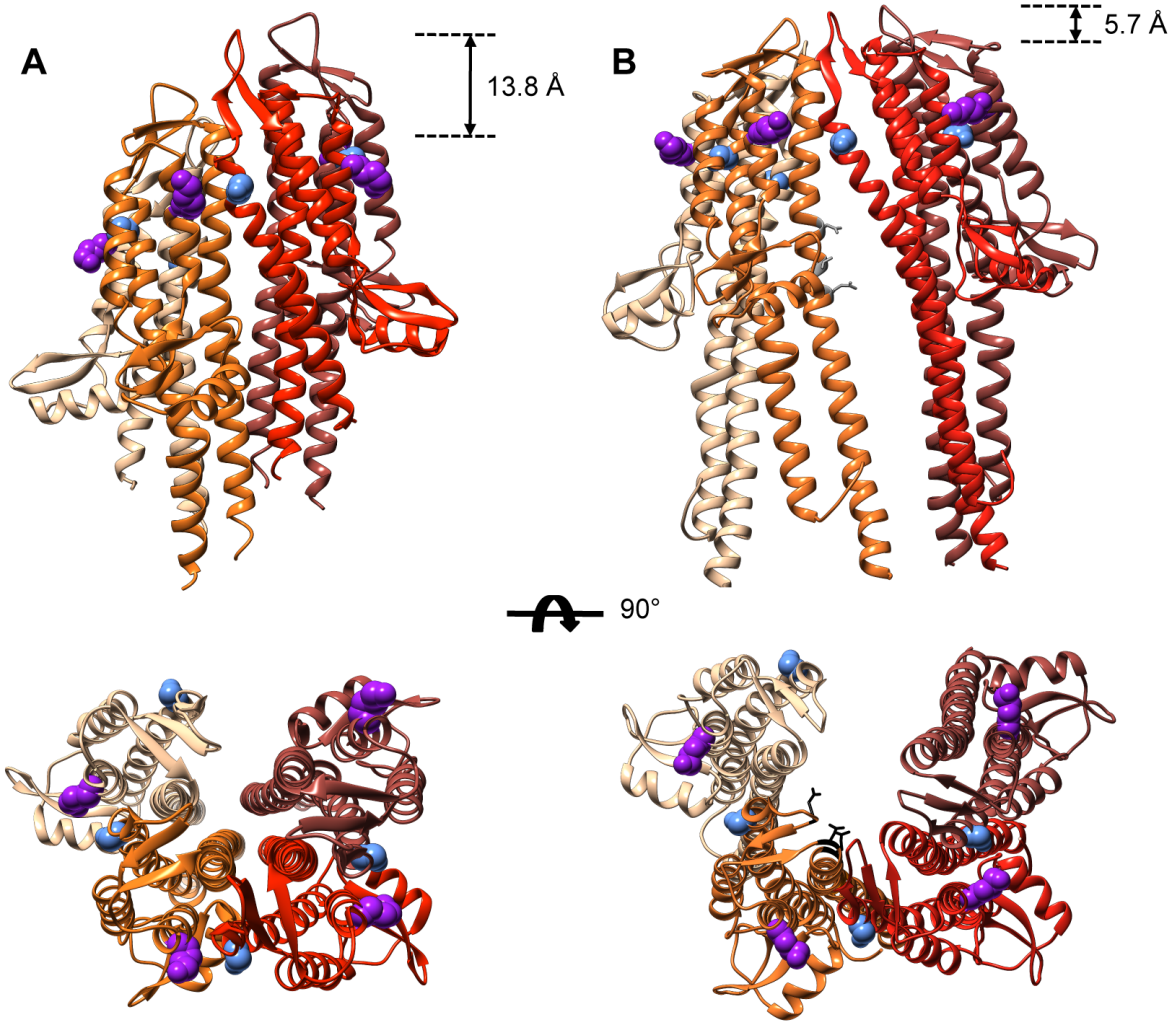
Comparison of crystallography and EM derived models of IpaD within the tip complex.A. Side view (*top*) and top view (*bottom*) of the tetrameric TC built from a crystal structure of dimeric IpaD lacking its N-terminal domain (PDB ID 2j0n; Johnson *et al*., 2007). To create the tetramer, the dimer was rotated 128° about the vertical axis and translated 8.6 Å in the Y-axis. The original dimer and shifted dimer were combined to form the TC model.B. Side view (*top*) and top view (*bottom*) of the tetrameric TC model built from the docking of four copies of a partial IpaD crystal structure (derived from PDB ID 2j0o (Johnson *et al*., 2007), molecule A as outlined in the main text) into the wild-type density map. The subunit colouring used is lightest for IpaD_β_ to darkest for IpaD_ε_. The blue spheres represent amino acid 173 and the purple spheres represent amino acid 258. In *grey* are the locations of weak mutations and in black are those for the strong mutations that lead to CR-insensitivity. The height differences between the lowest and highest subunits are displayed for each model.

The differences between our previous model of IpaD in the TC (Johnson *et al*., 2007; Blocker *et al*., 2008), which does not fit in our maps, and the model that emerges here from the docking of IpaD subunits to the wild-type map and from our crosslinking studies are illustrated in Fig. 6. In both models the subunits have the same relative orientation: C-terminal helices lining the central channel and C-terminal globular domain facing outwards. However, the subunits are much further apart in the new model (Fig. 6B), allowing for the presence of both a cavity and a perpetual pore within the TC. This fits with our crosslinking experiments, which indicate that amino acids 173 and 258 of neighbouring IpaD subunits in the TC are not close enough to each other to form disulphide bonds. These experiments are further supported by our attempts to make other amino acids, adjacent to 173 and 258 along the α3 and α6 helices, react chemically (Supporting Table S3). The latter indicate that those nearest to 173 and 258 generally interact best, but do so only in the presence of the long-armed crosslinker. In the new model the Cβ-Cβ distances between these amino acids for IpaD_β_ to IpaD_ϒ,_ IpaD_ϒ_ to IpaD_δ_, IpaD_δ_ to IpaD_ε_ are 13.5, 9.9 and 10.8 Å respectively. These distances are within the estimated, flexible length (8–15 Å) of the crosslinker we used (Green *et al*., 2001). In addition, the equivalent IpaD_α_ to IpaD_β_ distance is 14.2 Å while that from the IpaD_ε_ to IpaD_α_ pair is 21.8 Å, suggesting that the homopentamers in this heterogeneous population are not crosslinked at the IpaD_ε_ to IpaD_α_ interface.

The TC is also flatter than previously thought (Fig. 6A and B, *top*). This is unlikely to be an image alignment artefact given the definition obtained for each subunit and how well an IpaD crystal structure can be docked into individual subunit densities. As a result, the subunits in the new model do not rise as steeply along the needle axis as would be expected if they were following the needle helical parameters. Indeed, each IpaD subunit interacts with the needle subunit below it at a different height (Fig. 4C), with the higher one, IpaD_ε_, inserting most deeply into the trough between two adjacent MxiH subunits. This is likely caused by the inhomogeneous arrangement of the subunits and TC asymmetry (Figs 4A and 5B). At this resolution, we cannot tell what causes this asymmetry although we suspect it results from domain rearrangements specific to each IpaD subunit.

### The spatial arrangement of IpaD subunits in the TC is different from that of all other models

Importantly, although our crosslinking data allow us to fit four or five IpaDs within our TC maps, they do not support the divergent orientations or conformations of SipD/IpaD subunits suggested to exist at the TC by other groups (Lunelli *et al*., 2011; Epler *et al*., 2012). An additional model was recently proposed (Rathinavelan *et al*., 2014), based on solution NMR paramagnetic relaxation enhancement (PRE) measurements of the interaction of an N-terminally truncated SipD with full-length PrgI, monomerised by two C-terminal point mutations (Poyraz *et al*., 2010). This work claims to identify two binding sites for PrgI on opposite faces of the SipD coiled-coil, using signals from spin label insertion at only four different sites in SipD. As PRE signals were not enhanced with any particular PrgI amino acid(s), the location of the binding sites on PrgI could not be defined. Nevertheless, the authors extrapolated their findings to dock five full-length SipDs at the tip of the atomic model of the PrgI needle proposed by Loquet *et al*. (2012), along the helical rise of the needle. Whilst this model resembles our initial one (Johnson *et al*., 2007; Blocker *et al*., 2008) and is not as grossly incompatible with our new model as those proposed by Lunelli *et al*. (2011) and Epler *et al*. (2012), we cannot assess its validity against our crosslinking data because no pdb coordinate file was released for it. Yet, having docked the SipD subunits as deep into the troughs between two adjacent MxiH subunits, the authors then have to position the N-termini of the SipD subunits pointing outwards at right angles from the needle. However, we do not see such densities in our maps and when the IpaD subunits are docked higher up in our model, there is spare density within our maps to accommodate their N-termini parallel to the needle. We note also that by far the strongest PRE signal observed was with SipD_D136_ (IpaD_A136_), which is located at beginning of helix α3, at the base of the coiled-coil and very close to all MxiH heads in our model. Curiously, this residue was then not used in the two-time-point PRE experiments performed to define the PrgI binding sites on SipD.

Finally, there are issues with model validation. All three other reports (Lunelli *et al*., 2011; Epler *et al*., 2012; Rathinavelan *et al*., 2014) include mutagenesis data as support for the validity of the highly divergent models proposed. However, all mutants made lead to loss-of-function phenotypes, e.g. loss surface localisation, constitutive secretion and/or loss of the ability to invade host cells. Evidently, this may occur due to loss of IpaD/SipD binding to the needle or other TC subunits. However, they may also occur upstream, due to protein misfolding. The IpaD mutants used in our cysteine-mediated crosslinking experiments have wild-type function and these experiments also yield key information on subunit distances and packing.

Ultimately, only higher resolution models of the native TC/needle interface will fully resolve how the TC is organised and connected to the needle. In principle, cryoEM analysis using the image-processing scheme developed here could improve the resolution of the 3DR obtained. However, in practice this is limited by the poor contrast of micrographs obtained in this imaging mode, the small size and lack of surface features of the TC. Indeed, our own attempts using such a data set have so far failed at the initial steps: precise identification of the needle tip during particle selection and accurate translational alignment of the particles.

### Consequences of newly proposed TC structure for its function

We propose that the TC's symmetry mismatch with the needle and its intrinsic asymmetry, two physical properties which are often used for biological mechanosensing because they put protein complexes in metastable states (Moody, 1999), make it perfectly poised for a conformational change upon physical contact with the host membrane (Campbell-Valois *et al*., 2014). The relevance of the flatness of TC top for translocon formation in the host membrane also remains to be investigated. However, since the translocon is likely to be planarly symmetrical (to lie within a planar membrane bilayer), we hypothesise that a flatter TC promotes the formation of the translocon by acting as an adapter between the helical needle and a planar translocon.

Finally, we recently identified point mutations in the upper part of the C-terminal helix of IpaD that lead to host cell and CR-insensitivity without constitutive secretion, indicating that they affect the ability of IpaD to signal downwards from the TC (Roehrich *et al*., 2013). All these mutations represent changes from polar to hydrophobic amino acids. The three weaker mutants N292, T296 and Q299, are located half-way down the C-terminal helix and in our new model they point 90° away from the TC channel (Fig. 6B, *top*). These may therefore affect signal transduction via changing the conformation of IpaD's C-termini, as previously proposed (Roehrich *et al*., 2013). The four stronger ones line the channel, with three lying very near top of the TC (N186, N273 and Q277), surrounding its central pore (Fig. 6B, *bottom*). Our new data therefore reveal that these could be involved in structural rearrangements of subunits, in particular at the interface with IpaB, for activation and signal transduction to the IpaD C-termini.

### Relevance of new TC model to therapeutics

The epitopes of disease-protective antibodies against LcrV and PcrV, IpaD homologues in *Yersinia* and *Pseudomonas* species respectively, have been mapped to the C-terminal globular regions of these proteins, corresponding to helix α4 of IpaD (Fig. 2A; Sato and Frank, 2011). This helix is surface exposed and facing the host cell in our model, but not in that of others (Lunelli *et al*., 2011; Epler *et al*., 2012). Therefore, antibody binding to this domain could inhibit host cell contact sterically and/or inhibit host cell sensing via inhibition of as yet unidentified conformational change(s). Interestingly, the only sequence variation within IpaD between different *Shigella* species lies precisely within or near this helix, and at surface exposed sites (Supporting Fig. S9), suggesting it may be under selective pressure to escape immune recognition in this organism as well.

Recently, antibodies to IpaB were shown to correlate with protection against shigellosis (Wahid *et al*., 2013). This independently supports the notion that this protein is exposed at the bacterial surface and functionally important at this location before host cell contact and invasion. The location of the protective epitopes against IpaB remains to be determined, although from our work and the findings on IpaD homologues, we propose they will map between the alacoil region and the C-terminal helix of IpaB, in an area which probably represents the upper bulge(s) seen in the larger molecule within the WT and *mxiH*_Q51A_ TC reconstructions.

## Experimental procedures

### Bacterial culture

*S. flexneri* strains were maintained and selected on CR agar plates (Meitert *et al*., 1991) and grown at 37°C in trypticase soy broth (Becton Dickinson) supplemented with antibiotics where appropriate (100 μg ampicillin ml^−1^, 50 μg kanamycin m^−1^, 10 μg chloramphenicol ml^−1^ and 5 μg tetracycline ml^−1^).

### Mutant construction

Strains and plasmids used are listed in Supporting Table S1. All primers used are listed in Supporting Table S2 and all constructions were verified by DNA sequencing (Eurofins).

### 

#### Double knockout mutant background strains

The generation of the Δ*ipaB* Δ*ipaD* mutant was described (Roehrich *et al*., 2013). The Δ*mxiG* Δ*ipaD* mutant was generated using the λ red system (Datsenko and Wanner, 2000). Briefly, a tetracycline resistance cassette with 50 bp flanking regions homologous to the *ipaD* gene was amplified from strain TH2788 (Frye *et al*., 2006) using the primers ipaD_KO_tetF and ipaD_KO_tetR. The *ipaD* gene was exchanged for this cassette in Δ*mxiG* strain SF703 (Allaoui *et al*., 1995) as previously described (Martinez-Argudo and Blocker, 2010). To generate strain Δ*mxiG* Δ*ipaB*, the same procedure was used to replace the *ipaB* gene in SF703 using the primers ipaB_KO_tetF and ipaB_KO_tetR. To construct strain Δ*mxiH* Δ*ipaD*, the *ipaD* gene was exchanged for a tetracycline cassette in Δ*mxiH* strain SH116 (Blocker *et al*., 2001) following same procedure (using the primers ipaD_KO_tetF and ipaD_KO_tetR). To make the Δ*mxiG* Δ*mxiH* strain, a kanamycin resistance cassette with 50 bp flanking regions homologous to the *mxiG mxiH* genes was amplified from plasmid pKD4 (Datsenko and Wanner, 2000) using the primers mxiGH_KO_F1 and mxiH_KO_R1. The *mxiG* and *mxiH* genes were simultaneously exchanged for a kanamycin cassette in the wild-type strain M90T following the same procedure.

#### mxiH mutants for EM

The Δ*mxiG* Δ*mxiH* strain was complemented with pSZ1 pBAD::*mxiG* expressing N-terminal 6 × His-tagged MxiG (Zenk *et al*., 2007) and pACT3::*mxiH* carrying wild-type or mutant *mxiH*_,_ amplified from previous constructs (Kenjale *et al*., 2005) using primers listed in Supporting Table S2 and cloned using NdeI and HindIII sites.

#### Avidin-labelling mutants for EM

The 15-mer Avitag (GLNDIFEAQ**K**IEWHE) is biotinylated *in vivo* by the bacterial enzyme BirA. Binding of avidin to the biotinylated Avitags requires the tag and biotin molecule to be accessible. The selection of Avitag insertion sites was based on the atomic structure of IpaD and its predicted fitting on the needle tips (Blocker *et al*., 2008). In particular, the N-terminal H1 and H2 α-helices form a two-helix bundle proposed to shift outwards upon needle binding, hence predicting an external orientation that would be surface accessible (Johnson *et al*., 2007). The Avitag was therefore inserted right before this N-terminal domain, after H39. The full atomic structure of IpaB is not available, but its overall fold and mode of needle binding may be similar to that of IpaD (Johnson *et al*., 2007; Roehrich *et al*., 2010). Therefore the Avitag was inserted into IpaB at A33, after the known secretion signal and 1^st^ chaperone-binding region of IpaB (Lunelli *et al*., 2009; Lokareddy *et al*., 2010). To further aid accessibility to BirA and then avidin, spacer sequences (LGTRGS) were inserted either side of the Avitag.

Insertion of the Avitag into IpaD was conducted via a two-step PCR reaction. Two PCR products were generated using primer sets ipaD_NdeI_F1 & avitagD_R1 and ipaD_EcoRV_R1 & avitagD_F1. In a second PCR reaction, the products from the first two PCR reactions were used as templates and primers ipaD_NdeI_F1 + ipaD_EcoRV_R1 were used for amplification. The resulting product was restricted with enzymes NdeI and EcoRV and cloned into expression plasmid pWPsf4D (Picking *et al*., 2005). The resulting *ipaD*_avitag construct thus encodes AA39-SPACER-AVITAG-SPACER-AA40. To allow for the affinity purification of NCs with IpaD_avitag TCs, *ipaD*_avitag was cloned into plasmid pIMA202. Previously, *mxiH* had been cloned downstream of *mxiG* into plasmid pSZ1 (pBAD::*mxiG* with N-terminal 6 × His-tagged MxiG; Zenk *et al*., 2007), resulting in the plasmid pIMA202 and the generation of restriction sites XmaI and ClaI either side of *mxiH*. These restriction sites were used to exchange *mxiH* with the *ipaD*_avitag construct, giving rise to plasmid pBAD::*mxiG ipaD_avi_*. The resulting plasmid was transformed into the Δ*mxiG* Δ*ipaD* strain, producing strain *ipaD*_avi_.

The strategy for inserting the Avitag into IpaB was similar. A two-step PCR reaction was used to produce a fragment encoding the sequence AA33-SPACER-AVITAG-SPACER-AA34. The fragment contained restriction sites HindIII and PstI at either end, which were used to clone the fragment into expression plasmid pUC19, resulting in *ipaB*_avitag. Again, *ipaB*_avitag was cloned into pIMA202 giving rise to pBAD::*mxiG ipaB_avi_*. The resulting plasmid was transformed into the Δ*mxiG ipaB* strain, producing strain *ipaB*_avi_.

#### ipaD_C322S_ background for cysteine-crosslinking

Single mutation C322S was introduced into *ipaD* via PCR using ipaD_EcoRV_for/ipaD_C322S_rev as primers and pWPsf4D (Picking *et al*., 2005) as template. The resultant PCR product was cloned back into pWPsf4D via EcoRV and PstI sites, giving rise to pUC18 *ipaD*_C322S_, which was transformed into Δ*ipaD* to obtain strain *ipaD*_C322S_, which is denoted as *ipaD* subsequently throughout.

#### Cysteine mutants for cysteine-crosslinking

Both *ipaD* single and double cysteine mutations were introduced via two-step PCR. For instance, to obtain single cysteine mutant *ipaD*_V170C_, 5′ and 3′ fragments of *ipaD* were amplified from pUC18 *ipaD*_C322S_ using the primer pairs ipaD_EcoRV_for/ipaD_V170C_rev and ipaD_V170C_for/ipaD_PstI_rev, respectively. In the second step, using the primer pair ipaD_EcoRV_for/ipaD_PstI_rev, the mixture of 5′ and 3′ fragments was used as the template to generate *ipaD*_V170C_, which was then cloned back into pUC18 *ipaD*_C322S_ via EcoRV and PstI sites. The resultant plasmid pUC18 *ipaD*_C322S/V170C_ was used to obtain strain *ipaD*_C322S/V170C_, which is then denoted as *ipaD*_V170C_. To obtain double cysteine mutants, pUC18 *ipaD*_C322S_ containing a single *ipaD* cysteine mutation was used as template in the first step PCR.

When both pACT3 containing *mxiH* and pUC18 containing *ipaD* were transformed into Δ*mxiH* Δ*ipaD* bacteria, *ipaD* expression was inhibited by LacI, encoded on pACT3, which bound to the *lac* operator of pUC18. To overcome this, we altered the pUC18 *lac* operator sequence to TGTGTGGAATT**A**T**TGTTA**GA**C**AA**T**AATTTCACACA, making it a nearly constitutive *lac* operator (O^c^), by ordering a 616 bp DNA fragment (Eurofins) covering the pUC18 *lac* operator region and the start of *ipaD*. This fragment was then cloned back into the appropriate pUC18 based *ipaD* mutant plasmids (see end of Supporting Table S1) using the SapI site of the plasmid and the EcoRV site within *ipaD*. The corresponding bacteria had normal expression of IpaD in the presence of 30 μM IPTG.

### Mutant validation and growth condition optimisation

Total level of protein expression, Ipa protein leakage and CR induction were determined as previously described (Martinez-Argudo and Blocker, 2010). Results presented are representative of at least two independent experiments.

Contact hemolysis of sheep red blood cells was performed as described previously (Blocker *et al*., 1999), all samples were tested in triplicate, and data presented are average results from three independent experiments.

HeLa cell invasion assays, also known as gentamycin protection assays, were performed as previously described (Shen *et al*., 2010).

For each mutant, the same number of exponentially growing bacteria (OD_600_ approximately 1), adjusted via optical density at 600 nm, was used in each assay.

#### mxiH mutants for EM

NCs were purified from 250 ml bacterial cultures from bacterial strains *mxiH_Q51A_*, *mxiH_P44A_* and *mxiH*_*P44A*+*Q51A*_ using the modified NC purification protocol below. Cultures were grown to mid-exponential phase with the addition of 15 μM (*mxiH_Q51A_* and *mxiH_P44A_*) and 90 μM (*mxiH*_*P44A*+*Q51A*_) IPTG to induce different levels of MxiH production. This was done to enhance expression of some needle mutants showing polymerisation defects until they allowed generation of NCs with needles of wild-type length.

#### Avidin-labelling mutants for EM

The concentration of arabinose required to achieve wild-type levels of IpaB_avitag and IpaD_avitag in TCs was established by the addition of varying levels of arabinose during growth. Analysis of the secretion of Ipa proteins through the T3SS, both during to overnight leakage and in response to CR, followed by invasion assays, was used to assess the TC composition of the mutants with respect to wild-type. Then, conditions for biotinylation and avidin-binding were optimised, using Western blot with peroxidase conjugated to streptavidin (from *Streptomyces avidinii*; Sigma-Aldrich) and a gel shift assay (see Supporting Information), respectively.

#### FACS analysis

Exponential phase bacteria (OD_600_ approximately 1) were washed twice in PBS, centrifuged at 6000 g for 3 min and resuspended at OD_600_ = 1 in PBS. Cells were fixed with 2% w/v paraformaldehyde (PFA), washed and resuspended in PBS containing 0.006% w/v azide. 6 × 10^6^ bacteria (20 μl) were immunolabelled with 20 μl of primary antibody in 0.5% w/v BSA in PBS (PBS-BSA), for 30 min at room temperature, washed with 1 ml PBS-BSA and recentrifuged as above. Pellets were resuspended in 20 μl of PBS-BSA containing secondary antibody and incubated as above, then washed as before and resuspended in 1% PFA and kept at 4°C in dark until use. Bacteria were immunolabelled with 100 ng of anti-IpaB (mouse monoclonal H16; Barzu *et al*., 1993), 200 ng anti-IpaD (470, a rabbit polyclonal antiserum raised, by Eurogentec, and affinity purified against IpaD_15–322_; Harlow and Lane, 1988) or 25 ng anti-MxiH [purified IgG (Harlow and Lane, 1988) from SK2102, a rabbit polyclonal antiserum raised by Eurogentec against native purified long needles]. One hundred nanograms of Biotin-SP-conjugated anti-mouse IgG (115-065-003, Jackson ImmunoResearch) followed by 100 ng of Streptavidin-PE-Cy5 conjugate (SA1006, Caltag Lab) were used to enhance H16 detection. Anti-IpaD and anti-MxiH were detected using anti-rabbit IgG-PE (P8172, Sigma) used at 1:20. A Canto II flow cytometer (BD Biosciences) running Diva 6.1.2 software was used to acquire at least 1 000 000 counts for each sample and the data were analysed with FlowJo v9.6.4. Excel was used to generate mean counts of bacteria-specific fluorescence signals.

### Electron microscopy

#### Purification of intact NCs with mutations in MxiH

NCs were affinity purified from strains with Δ*mxiH* Δ*mxiG* background harbouring pBAD::*His_6_mxiG* and plasmid pACT3::*mxiH*, encoding MxiH point mutations of interest, using a protocol modified from Zenk *et al*., 2007. Briefly, 250 ml cultures of Trypticase Soy Broth (TCSB, BD) were inoculated and grown to mid-exponential phase (OD_600_ = 1), in the presence of 0.02% arabinose and between 15 to 90 μM IPTG, depending on the strain, to upregulate *mxiH* expression. Bacteria were harvested by centrifugation (10 min, 2500 *g*, 4°C) and washed with 100 ml phosphate buffered saline (PBS) before pelleting again. Bacterial pellets were resuspended in 12.5 ml 0.5 M sucrose, 100 mM Tris pH 8. Spheroplasts were formed by the addition of 125 μl 100 mM EDTA and 2 ml 10 μg ml^−1^ lysozyme (Wako) upon incubation on ice (1 h, constant stirring). Spheroplasts were lysed by addition of 2.5 ml 10% n-Dodecyl-β-D-Maltopyranoside (DDM, Affymetrix). DNA was removed by adding 50 μl deoxyribonuclease I (DNase I, Sigma), 2.5 ml 100 mM MgSO_4_ and cell debris pelleted by centrifugation (20 min, 21 000 *g*, 4°C). The supernatant was removed and NCs were pelleted by centrifugation (2 h, 94 000 *g*, 4°C). The resulting pellet was resuspended in 10 ml resuspension buffer (5 mM imidazole pH 8, 150 mM NaCl, 10 mM Tris pH 8, 0.5% w/v DDM, 1 small EDTA-free protease inhibitor tablet, Roche) by passing it through a 22-gauge syringe and the resuspension incubated with 400 μl Ni-NTA agarose beads (Qiagen) for 4–16 h (4°C, with constant mixing). Beads were pelleted by centrifugation (5 min, 1000 *g*, 4°C), the supernatant removed and beads were washed with 15 ml wash buffer 1 (50 mM imidazole pH 8, 150 mM NaCl, 10 mM Tris pH 8, 0.1% (w/v) N-Lauroylsarcosine sodium salt (Sigma), 1 small EDTA-free protease inhibitor tablet), followed wash buffer 2 (50 mM imidazole pH 8, 150 mM NaCl, 10 mM Tris pH 8, 0.2% w/v DDM, 1 small EDTA-free protease inhibitor tablet). Beads were pelleted (5 min, 1000 *g*, 4°C) and the supernatant was removed, leaving just enough buffer to cover the beads. NCs were eluted by addition of 55 μl 1 M imidazole pH 8 and incubation on ice for 30 min, with agitation every 5 min. Following centrifugation (5 min, 1000 *g*, 4°C), the NCs in the supernatant were collected using a Nanofil 100 μl glass syringe with a 26-gauge needle (World Precision Instruments).

#### Avidin labelling of IpaB and IpaD in purified NCs

NCs were purified in a similar manner, but with the following modifications. 250 ml bacteria were grown to mid-exponential phase in the presence of 0.05 mM biotin (Sigma, TLC grade). Arabinose concentrations were increased to 0.08% for *ipaB*_avi_ and 0.12% for *ipaD*_avi_ in view the functional validation of the strains described above. After incubation with the Ni-NTA agarose beads, the beads were transferred to 2 ml microcentrifuge tubes. Avidin (Sigma, BioUltra) was added to the tubes (300 molar excess for *ipaB*_avi_ and 500 molar excess for *ipaD*_avi_, as estimated from the number of bacteria used for each preparation and assuming 100 NCs per bacterium) and incubated overnight at 4°C. Beads were washed and eluted as before, but with the number of washes for Wash 1 increased to 10.

#### EM of purified NCs

Purified NCs were prepared for EM using a modified Valentine staining technique (Valentine *et al*., 1966), which was found particularly valuable for allowing effective and uniform dispersal and staining of these detergent containing samples. Thin sheets of mica were coated with a thin layer of carbon by evaporation. The mica sheets were cut into 5 × 3 mm rectangles and partially submerged into 100 μl of 0.01% (w/v) DDM until most of the carbon detached from the mica. Once detached, the mica was removed and gently blotted dry using filter paper (Whatman). The carbon was then lifted on the mica support and partially floated on a 5 μl drop of sample for 10 s, allowing the sample to disperse between the mica and carbon. Excess sample was removed by blotting and then washed by partial floatation in a 20 μl drop of ddH_2_0 for 10 s. The mica was used to lift the carbon from the ddH_2_O drop and immediately dropped into a 40 μl drop of 1% uranyl acetate, allowing the carbon and mica to detach completely and the carbon to float on the drop surface. A 300 mesh copper grid (Electron Microscopy Sciences) was gently placed on top of the drop, coating the grid with the carbon film. Girds were blotted dry and kept desiccated until use.

Electron micrographs were recorded on a FEI Tecnai T20 transmission electron microscope (TEM), operating at 200 kV. Images were acquired at a nominal magnification of 50 000X, employing a low-dose system operating at ∼10 e/Å^2^. Images were recorded on a FEI 4k × 4k Eagle CCD camera controlled by TIA software. During focusing, the 1st zero of the contrast transfer function (CTF) was set at ∼19 Å, removing the need for later CTF correction. Using the major TMV layer line spacing at 22.92 Å (Kendall *et al*., 2007) in Fourier space, this magnification was calculated to give 2.12 Å/pixel resolution.

#### 3D reconstruction of TCs from EM micrographs

Needles from individual NCs were manually selected and saved as individual helix images of 120× ∼200 pixels using helixboxer in EMAN2. Care was taken to set the needle and tip positions (central and vertical and 44 pixels from the top of the image, respectively) similarly for all images using Rulers (Mac App Store). Individual images were trimmed to exactly 120 × 200 pixels using a dedicated MATLAB program and then stacked into a SPIDER file, ramped and normalised in MATLAB. Images were precisely aligned translationally using a dedicated MATLAB program (2Dalign) described in the Supporting Information. This programme had to be developed because other existing programmes (such as SPIDER routines and EMAN2 refine2D) failed to align the particles effectively, possibly because of their small size and/or the lack of contrast at the distal tip. Precise translational alignment is an absolute pre-requisite for obtaining class averages with clear helical patterns in the needle portion, which itself is required for assigning an azithumal angle to each class average by matching to projections of a needle model. Inconsistent tip heights at any point leads to misassignment of azimuthal angles. Therefore, translational alignment was optimised here and up until the refinement stage using bespoke programmes.

##### 2D classification

Image data set were band-pass filtered to 8–200 Å using EMAN (Ludtke *et al*., 1999) (proc2d command) and further processed in SPIDER (Frank *et al*., 1996). The number of classes requested was selected to include approximately 20 particles per classes so as to give a readily detectable needle helical pattern in real and Fourier space. For each data set, Eigenvectors were computed in SPIDER using CORAN (CA S) and reconstituted using CA SRE. This allowed us to access the helical pattern of each vector. Classes were created by *k*-means clustering (CL KM) using only those vectors that showed strong helical pattern. Class averages where created by summation of images in each class (AS).

##### 3D reconstruction

Classes were corrected for in-plane rotation by matching to projections of a 3D map of the *Shigella* needle obtained using negatively stained data (Cordes *et al*., 2003) using AP SH in SPIDER. *x*- and *y*-shifts are first corrected by calculating the cross-correlation of classes with a class average where the needle information has been blurred (for this a class average is selected, mirrored across its y-axis and then summed with the original image), followed by manual correction using a dedicated program called tkmanualshift.py, also described in the Supporting Information.

An initial 3D map is generated in SPIDER in three steps. First, the azimuthal orientation of each class is determined by projection matching (AP SH) to projections of the Cordes *et al*. (2003) model at 2–4° intervals, depending on the number of classes obtained for each data set. For all AP SH steps, the radius of the search range was 50 pixels from the centre of the image, with a step size of 1 pixel. The BP 3F function is used to back-project the classes into an initial model. A new reference volume is created by imposing the previously determined helical symmetry of the needle (axial rise 4.3 Å, axial rotation 64°; Fujii *et al*., 2012) using h-impose (Egelman, 2000) to only the needle segment of the previous model. The needle segment was selected as all density 120 Å below the distal tip of the needle, because the TC was clearly delineated in the class averages (Supporting Fig. S6) and 120 Å would generously accommodate the height of an IpaD subunit (Johnson *et al*., 2007). The final 3D map is generated by iterating the procedure 5 times from the projection-matching step onwards. For all BP 3F steps, the projection radius was 50 pixels.

##### Estimation of resolution

Each data set was divided into equal and odd particle number groups. The reconstruction parameters calculated previously were applied to each data set half, creating two 3D maps (BP 32F). The FSC between the two new reconstructions was determined using the SPIDER function FSC, with FSC curves produced in Excel. The resolution of each reconstruction was determined as the point at which the FSC dropped below 0.5.

##### Map refinement for misalignment within TC subunits within TC from the *ΔipaB* strain

The initial electron density map of the TC in NCs from Δ*ipaB* (not shown) was used to generate a new reference by removal of density due to azimuthal misalignment of subunits. This was identified at the interface between the lowest and highest IpaD subunit. This new map was projected every 2° to create 180 reference images. Then, the images within the original unaligned stack used for the initial reconstruction were shifted using the alignment parameters derived from that 3DR. The shifted images are then grouped based on their azimuthal angle with each covering 2°. Averages were generated for each group and visually compared to the projections of the map, recording those where extra density was seen at the location described above. For each group Eigen analysis was conducted and the first ten Eigen images inspected. The Eigen images, which showed variance at the interface between the highest and lowest subunit were used to separate the images in each group into three classes. Images belonging to classes where extra density could be seen at this location were removed from the stack and the 3DR process was repeated.

##### Map refinement using raw data from TCs from mxiH_Q51A_

Density map refinement used a similar methodology to that described above. However, instead of using class averages, *x*- and *y*-shift correction was conducted on groups of individual images as opposed to class averages. After correcting images for in-plane rotations, images were randomly divided into 50 sets and corrected for x- and y-shifts using tkmanualshift.py. Eigenvectors and images were computed for the TC portions of each set. Within each set, the individual images are classified by *k*-means clustering using vectors that showed significant shifts in the *y*-axis. Then, each class was manually shifted to a reference using tkmanualshift.py. After classification, images were again randomly divided into classes and the procedure was iterated until minimal *y*-shifts were observed in the eigenvectors. Azimuthal orientations of the individual images were calculated by projection matching to the density map of the TC portion only, generated using the method described above.

All programs and scripts mentioned above, along with instructions and test data sets, are available from CCPEM (http://www.ccpem.ac.uk/download.php) and at DOI 10.5523/bris.1trtuj35bwn5410ibhinps47ag.

#### Statistical analysis of avidin binding to purified NCs

This is described in the Supporting Information.

### Assessment of cysteine-crosslinking/disulphide bond formation in needle tip located IpaD

Mutants were initially screened by shearing long needles from the bacterial surface. Exponential phase bacteria (OD_600_ approximately 1), where *mxiH* overexpression had been induced with 200 μM IPTG, were harvested. 1.2 ml of bacteria concentrated to OD_600_ = 12 were exposed to 60 μM of freshly prepared oxidiser sodium tetrathionate (O) or membrane impermeable crosslinker 1,8-bismaleimidodiethylene glycol (BM(PEG)_2_) in PBS pH 7.4 for 15 min at 37°C. The samples were quenched by addition of 3 mM freshly prepared L-cysteine. Needles were sheared off the bacterial surface using a 1 ml syringe connected to a 26 g needle (15 up-down strokes), 1 ml supernatants were collected after centrifugation (16 000 *g*, 20 min, 4°C), precipitated by adjustment to 13% w/v trichloroacetic acid (TCA) and washed twice with cold acetone. Pellets were resuspended in non-reducing (O) or reducing SDS-PAGE loading buffer (BM(PEG)_2_).

Sets of Cys mutants demonstrating the strongest signals were also tested in other ways. First, long needles were precipitated using NaCl_2_ and PEG6000, instead of TCA (Cordes *et al*., 2005). Second, NCs, instead of sheared needles, were prepared as described above but with minor modifications and on a small scale. Briefly, 30 μM BM(PEG)2 or oxidiser (O) treated bacteria were washed in PBS, then quenched in 1.5 mM L-cysteine and washed again in cold PBS, resuspended in 0.9 ml 0.5 M sucrose, 100 mM Tris, 1.37 mM EDTA, pH 8 and spheroplasted by adding 125 μl of 10 mg ml^−1^ lysozyme. After 30 min incubation at room temperature, cells were lysed with 200 μl of 10% DDM containing EDTA-free protease inhibitors, then 250 μl of 100 mM MgSO_4_ and 5 μl of DNase. After 5 min incubation at room temperature, the samples were centrifuged at 21 000 g at 4°C for 15 min; the supernatants were collected, further centrifuged at 94 000 g at 4°C for 30 min. The pellets were resuspended in non-reducing (O) or reducing SDS-PAGE loading buffer (BM(PEG)_2_).

For all these experiments, samples equivalent to 3 ml culture OD_600_ 1 were loaded into SDS-PAGE and analysed by Western blot using either a polyclonal anti-IpaD rabbit serum (Menard *et al*., 1993) -for samples derived from strains expressing long needles- or -for samples of crude NC preparations- the anti-IpaB and IpaD antibodies mentioned in the FACS section above. Primary antibodies were detected using secondary antibodies raised in goat and coupled to Alexa 680 (gαr IgG, A21109, Invitrogen) or to DyLight 800 (gαm IgG, 35521, Thermo Scientific) fluorophores. The membranes were then visualised by using an Odyssey infrared imaging system (LI-COR Biosciences).

### Docking and molecular modelling of IpaD subunits within TCs

Maps were normalised against the wild-type map using the EMAN function Volume. For each map, the mass/density threshold was calculated using a mass estimate of 675 717 Da calculated as 1 IpaB, 4 IpaD and 50 MxiH molecules, based on the number of individual protrusions seen in the ‘unrolled’ 3D map. This gave thresholds of 0.00688, 0.00876, 0.008560, 0.008050 and 0.007669 for wild-type, Δ*ipaB*, *mxiH_Q51A_, mxiH_P44A_ and mxiH*_*P44A*+*Q51A*_ respectively. The wild-type map was contoured to a level of 0.02 in Chimera using the 3-sigma criterion. However, increasing the contour level to 0.03 enhanced structural features in the map so this level was used for normalisation. The contour level for the other maps was calculated as (threshold of map*0.03)/0.00688. For the *mxiH_Q51A_* map, this gave a contour level of 0.037, which resulted in artefacts such as ‘holes’ in the needle portion. For this map, we decreased the contour level to 0.03, which resulted in better representation of the needle and thus TC.

The electron density map of the needle derived from our cryoEM work (EMD-5352; Fujii *et al*., 2012) was docked into each TC map manually, by identifying the position of the lowest TC subunit and aligning it with subunit insertion P1 in the needle. This was performed and optimised in Chimera (using default settings, except ‘in map simulated atoms, resolution’ set at 20Å; Pettersen *et al*., 2004). An N-terminally truncated and C-terminally modified IpaD crystal form (PDB code 2j0o, molecule A) was fitted into each TC density in Chimera. The molecules were fitted manually and one at a time until a total of 4 or 5 was reached. The fit of each IpaD was then optimised by inspection, with particular regard to the large bulges on the outside of TCs, to the tilt of each subunit and to contacts between neighbouring subunits. The cysteine-mediated crosslinking data were used to optimise subunit orientation. The models were checked for consistency with the crosslinking data by measurement of Cβ-Cβ distances between residues 173 and 258 of neighbouring IpaD subunits. This process was repeated until a self-consistent model was achieved. In all cases, the correlation coefficients obtained between the atomic models and the electron density maps (using the Chimera ‘fit in map function’) were greater than 85%.

## Accession codes

All electron density maps are available from the EMDB (wild-type, EMD-2801; *ΔipaB*, EMD-2802; *mxiH*_P44A_, EMD-2803; *mxiH*_Q51A_, EMD-2804; *mxiH*_P44A+Q51A_, EMD-2806; refined *mxiH*_Q51A,_ EMD-2805). The model of the four modified IpaD subunits generated from docking into the wild-type map is available from the PDB under accession code 4d3e and also linked to EMD-2801.

## Author contributions

AJB designed, funded and coordinated the research; IMA and MC prepared strains for EM analysis; MC functionally tested the strains and he and AJB optimized sample & grid preparation for EM; MW, JM and AJB acquired the EM data; TK tested procedures for classification and optimized alignment of classes and single particles in 2D; FM designed the 3DR procedure, which he implemented with MC, who also performed all classifications, class alignments and refinements; PM and TT performed the statistical analysis on the avidin-labeled TCs; DKS, ADR, GF, JB, and XL designed, constructed and validated strains for cysteine crosslinking; MP performed the affinity purification of the new anti-IpaD polyclonal antibodies; DKS, JB and GF performed the cysteine crosslinking experiments; RBS and MC performed the molecular dockings; KN provided guidance in EM data processing and overall interpretation of results; AJB, MC, DKS, and RBS and wrote the paper with input from all others.
