# Supplementary material for: Three-dimensional electron microscopy reconstruction and cysteine-mediated crosslinking provide a model of the type III secretion system needle tip complex
Source: Mol Microbiol. 2014 Nov 27;95(1):31–50. doi: 10.1111/mmi.12843 (PMC4539596; doi:10.1111/mmi.12843)
Supplement: Supplementary file 1 — Supporting Information [file mmi0095-0031-sd1.pdf]

SUPPORTING INFORMATION FOR

Three-dimensional electron microscopy reconstruction and cysteine-mediated crosslinking provide a model of the T3SS needle tip complex

Martin Cheung[1]*, Da-Kang Shen[1]*, Fumiaki Makino[2]*, Takayuki Kato[2]*, A. Dorothea Roehrich[1], Isabel Martinez-Argudo[1], Matthew L. Walker[3], Isabel Murillo[1], Xia Liu[1], James Brown[1], Gordon Frazer[1], Judith Mantell[4], Petros Mina[5], Thomas Todd[5], Richard B. Sessions[6], Keiichi Namba[2,7]** and Ariel J. Blocker[1]**

[1]University of Bristol, Schools of Cellular & Molecular Medicine and Biochemistry, BS8 1TD, United Kingdom; [2]University of Osaka, Graduate School of Frontier Biosciences, 565-0871, Japan; [3]MLW Electron Microscopy Consulting Launceston, Cornwall PL15 9BB, United Kingdom; [4]University of Bristol, School of Biochemistry, Wolfson Bioimaging Facility, BS8 1TD, United Kingdom; [5]University of Bristol, Bristol Centre for Complexity Sciences, Predictive Sciences Helpdesk, BS8 1TR, United Kingdom; [6]University of Bristol, School of Biochemistry, BS8 1TD, United Kingdom; [7]Riken Quantitative Biology Center, Osaka 565-0871, Japan.

*These authors contributed equally to this work

**Joint senior authors

This file contains:

Supporting Note (containing additional Experimental Procedures and Results)

Supporting Figures S1-7

Supporting Tables S1-4

Supporting References

EXPERIMENTAL PROCEDURES

Image processing

<u>TC 2D alignment algorithm (2Dalign)</u>

Needle tip images were more precisely aligned translationally using the dedicated MATLAB program described below.

Image averaging

First, an average of the images is computed, forming image A. We select a rectangular region of A where the needle is dominant, giving rise to average image $A(i_1{:}i_2,j_1{:}j_2)$, where pixel numbers i_1 and i_2 bound the rectangle in the *x* (vertical)-direction and j_1 and j_2 bound the rectangle in the *y* (horizontal)-direction.

2D correlation alignment algorithm

In 2D correlation, the set of images (I) are first averaged to form an average image $A(i_1{:}i_2,j_1{:}j_2)$ as detailed above. The 2D correlation algorithm then takes each image I and selects a rectangular area within I, creating a subimage $I(i_1+p{:}i_2{:}p,j_1+q{:}j_2+q)$, where p and q are two integer variables. The correlation between $A(i_1{:}i_2,j_1{:}j_2)$ and $I(i_1+p{:}i_2{:}p,j_1+q{:}j_2+q)$ is maximised by varying p and q. The 2D correlation between the average image A and a particular image I for any values of p and q is defined by

$$Correlation(A,I,p,q)=\frac{\sum_{i=i_1}^{i_2}\sum_{j=j_1}^{j_2}A(i,j)I(i+p,j+q)}{norm(A(i_1:i_2,j_1:j_2))norm(I(i_1+p:i_2+p,j_1+q:j_2+q))}$$

where norm denotes the Frobenius norm as defined by

$$norm(A(i_1:i_2,j_1:j_2))=\sqrt{\sum_{i=i_1}^{i_2}\sum_{j=j_1}^{j_2}A(i,j)A(i,j)}$$

Each image I is implicitly shifted by the optimal p and q for that image. This alignment algorithm is repeated for all images and a new

average A is computed. The whole process is then repeated many times until the average converges.

Image processing scheme

Images $I(i_1+p:i_2+p,j_1+q:j_2+q)$ are aligned to average an $A(i_1:i_2,j_1:j_2)$ by 2D correlation and the process iterated until the images converge. A correlation cut-off of 0.3 was set and images that fell below this value were discarded. Furthermore, images which required translations greater than 12 pixels vertically and 8 pixels horizontally were also discarded. Shifted images saw their shifted portion(s) replaced by the average grey value of the images at the final shift.

<u>Program for semi-automated realignment of TC classes and individual images (tkmanualshift.py)</u>

This program displays a graphical user interface, which has three views from left to right: a selectable reference, the difference between the reference and an image and the selectable image to be aligned to the reference. The program also displays two profile windows, which each contain 1-D projections of the reference (red) and image (yellow) in the X and Y direction, respectively. The user shifts the images to be aligned in the X and Y directions using the arrow keys, and iteratively evaluates the alignment using the difference image and by assessing the fit between the two 1D profiles.

Statistics

<u>Statistical analysis of avidin binding to purified NCs</u>

Determination of number of avidin molecules bound to each TC

Due to the observed variation in the position of the bound avidin within TC images, standard procedures of image classification could not be used. To surmount this, bound avidin was visualised by reconstituting each image using eigenvectors. The first 50 eigenvectors were calculated using CA S in SPIDER and reconstituted using CA SRE. We then reconstituted the original raw images using CA SR, selecting only the 25 strongest vectors. This had the effect of removing much of the noise from the raw data, allowing

us to directly visualise the bound avidin. As vectors corresponding to the avidin ranked highly, it was assumed that, by selecting 25 vectors, no information corresponding to the avidin was lost. Raw and reconstituted images were visually compared for avidin binding and, once the reconstitution process was found to be reliable, the reconstituted images were divided manually into classes corresponding to the number of bound avidin molecules. In addition, there were 238 images where the presence of 1 avidin could not be established with certainty due to the noisy nature of the data. We proceeded by considering the raw data and also an adjustment to the data analysis to take into account the ambiguous images.

Modeling approaches

Not wishing to make any assumptions as to specific binding mechanism of avidin binding to IpaD subunits, we modeled the binding process in two ways; (i) as a Poisson process and (ii) as a site exclusion binding process.

Derivation of the λ parameter in the Poisson process

In the context of the experimental setup the Poisson distribution is:

$$P(R = r) = p(r) = \lambda^r \frac{e^{-\lambda}}{r!}$$

where the *p(r)* is the probability of finding *r* bound molecules and λ represents the mean of the binding process. When *r=0*:

$$p(0) = e^{-\lambda}.$$

Rearranging for λ we have, $\lambda = -ln(p(0))$.

We can obtain the parameter λ using *R=0*. Due to the ambiguity of the generated experimental data *P(R=0)* can assume two values. If we remove the 238 ambiguous images from the total data then *P(R=0) = 5094/6782 = 0.7511* hence λ =0.2862. If we include the ambiguous data then *P(R=0) = 5332/7020 = 0.7511* hence λ =0.2750. Subsequently the values for λ were used to solve for the expected number of bound avidins in the respective totals using MATLAB.

Site exclusion heuristic formulas

We consider the number of available binding sites to be four, one for each of the available subunits. We consider the probability of binding to a TC

both as independent and proportional to the available bind sites. Thus, the probability of binding *P* is the probability of overall binding *p* multiplied by the site availability. Namely:

- When there are four sites available; P = p × 4/4.
- When one avidin is already bound; P = (p × 4/4) × (p × 3/4).
- When two avidins are already bound; P = (p × 4/4) × (p × 3/4) × (p × 2/4).
- When three avidins are already bound; P = (p × 4/4) × (p × 3/4) × (p × 2/4) × (p × 1/4).

For the unadjusted data *p=(1-5332/7020)=0.2405* and for the adjusted data (ambiguous images removed) *p=(1-5094/6782)=0.2489*. After calculating the probability of being bound under each of the four scenarios above we multiply the obtained probability *P* by the total number of images in the adjusted and unadjusted cases respectively. The result is the expected frequency of bound avidins.

RESULTS

Optimisation/validation of mutant strains used for TC analysis by electron microscopy

Avidin labeling strains

Concentrations of arabinose used

ipaD$_{avi}$ *and ipaB*$_{avi}$ strains were grown overnight with 0.02 and 0.08% arabinose. Δ*ipaB* and Δ*ipaD* were used as negative controls and WT as a positive control. The Ipa protein patterns of culture supernatants from both new strains, processed by SDS-PAGE and Silver staining, were very similar after growth with low or high arabinose concentrations. As the constitutive secretion phenotypes seen in Δ*ipaB* and Δ*ipaD* were strongly repressed at both 0.02% and 0.08% arabinose concentrations (not shown), significant TC functionality is restored under either condition. An arabinose concentration of 0.08% was subsequently used for overnight growth of both strains.

Such overnight cultures were sub-cultured and grown to mid-exponential phase, in the presence of 0.02 to 0.1 % arabinose. The induction of protein secretion through the T3SS was activated by addition CR as described in the Online Methods. Δ*ipaB* and Δ*ipaD* showed constitutive secretion of proteins prior to the addition of CR and a lack of response to CR, as expected(Veenendaal *et al.*, 2007). Silver stain analysis of proteins secreted following CR activation (Fig. S2 A,B) showed an increase in complementation in both mutants relative to wild-type as the arabinose concentration increased. An arabinose concentration of 0.08% gave near wild-type levels of complementation. This indicates that the proteins expressed via the plasmid constructs form functioning TCs. Therefore, at arabinose concentrations above 0.08%, the composition and morphology of TCs with IpaB_avitag or IpaD_avitag resembles that of wild-type.

To further test the TC functionality of the strains carrying the Avitag with respect to wild-type, we assessed their ability to sense and invade HeLa cells as described in the Online Methods. Both strains were grown overnight and then exponentially in the presence of 0.08% arabinose. Both strains retained the ability to invade HeLa cells, indicating the presence of functional TCs (Fig. S2C). The presence of IpaB_avitag in the TC led to a decrease in the invasion efficiency (to 64% of wild-type levels), although this was deemed acceptable. However, a large increase in the invasion efficiency was seen for strains expressing IpaD_avitag (to ~423% of wild-type levels). This hyper-invasive phenotype suggested possible incomplete complementation of $ipaD_{avi}$, which was leading to increased secretion of proteins through the T3SS before HeLa cell contact is made. Although the protein(s) responsible for mediating host cell adhesion is not known, increased adhesion would result in increased invasion(Roehrich *et al.*, 2010). To ascertain whether the increased invasiveness of the strain expressing IpaD_avitag was due to incomplete complementation, the invasion assay was repeated with increased levels of arabinose (Fig. S2D). Increasing the arabinose concentration to 0.12% substantially decreased the invasion efficiency, with levels approaching that of the wild-type. Increasing the arabinose concentration beyond 0.12%

did not lead to further improvement, so 0.12% arabinose was selected for exponential growth of *ipaD*$_{avi.}$

Optimisation of biotinylation

In *Shigella*, as in the closely related *E. coli*, biotin is converted to its active form (biotinyl-5′-adenylate) by the enzyme BirA(Wilson *et al.*, 1992), which also targets biotin to critical lysine residues within the Avitag. Intracellular levels of biotin are regulated by biotinyl-5'-adenylate in a negative feedback loop. Biotinyl-5'-adenylate targets BirA to an operator site within the *bio*A-BFCD operon thereby inhibiting synthesis of biotin. With intracellular biotin levels tightly regulated in this manner, it was important to determine whether biotinylation of our constructs was complete or whether addition of exogenous biotin would increase biotinylation. Indeed, maximal biotinylation of the constructs is critical in achieving quantitative avidin labeling.

To ascertain the degree of *in vivo* biotylation, *ipaD*$_{avi}$ and *ipaB*$_{avi}$ were grown to mid-exponential phase, with the concentrations of arabinose determined above. Cultures were grown in parallel with or without exogenous biotin at 0.05 mM, added during the exponential growth period. The degree of biotinylation was analysed by Western blot using peroxidase conjugated to streptavidin.

Both constructs allowed significant increases in levels of biotinylated avi-tagged proteins when exogenous biotin was added during growth to mid-exponential phase (Fig. S4A,B). For IpaB_avitag, significant biotinylation was seen in the absence of exogenous biotin. However, addition of exogenous biotin greatly enhanced biotinlyation. Biotinylation of IpaD_avitag was similarly enhanced by exogenous biotin, although to a lesser extent. Therefore exogenous biotin was added in all further experiments. We also titrated the amount of biotin added and found that 0.05 mM was optimal (not shown) and this was hence used throughout.

Optimisation of avidin binding

Having optimised arabinose and biotin concentrations, NCs were purified from *ipaD*$_{avi}$ and *ipaB*$_{avi}$, with NCs incubated with 500x and 300x molar excesses of avidin during purification, respectively, as described in the Online Methods. The efficiency of labeling was determined by the avidin shift

assay(Bueler & Rubinstein, 2008). Briefly, the affinity between avidin and biotin is so high (K_d = 10^{-15} M) that the interaction between the molecules remains intact even during SDS-PAGE. This results in an apparent increase is molecular weight of the target protein, visualised as a shift in band position by SDS-PAGE (often into the stacker). Boiling of the sample denatures avidin and releases the biotinylated protein. The degree of avidin labeling is therefore ascertained by comparing the levels of labeled protein detected before and after boiling. At the vast molar excesses of avidin used, we found that the protein labeling efficiency was nearly 100% for $ipaD_{avi}$ and approximately 80% for $ipaB_{avi}$ (Figure S4C,D).

Statistical analysis of avidin binding

A total of 7020 tip images, results of a single binding experiment, were analysed manually and the number of particles with different number of bound avidins counted: 1260 images with 1, 389 with two, 36 with 3 and 3 with 4 avidins, respectively. In addition, there were 238 images where the presence of 1 avidin could not be established with certainty due to the noisy nature of the data. We proceeded by considering the raw data and also an adjustment to the data analysis to take into account the ambiguous images, as shown in Fig. S4C.

Poisson distribution modeling

If the process of avidin binding to the IpaD subunits is purely random then this can be modeled as a Poisson process with mean $\boldsymbol{\lambda}$ where $\boldsymbol{\lambda}=0.2750$ or $\boldsymbol{\lambda}=0.2862$ for the unadjusted and adjusted data (as explained below). With $\boldsymbol{\lambda}=0.2750$ the expected frequency (rounded to integer) of finding one, two, three or four bound avidins per TC is 1466, 201, 18 and 2, respectively. With $\boldsymbol{\lambda}=0.2862$ the expected frequency is 1458, 208, 19 and 2 respectively. Both models follow the overall trend of the data very well (Fig. S4C).

Modeling using a site-exclusion heuristic

If we assume that avidin binding is independent but upon binding to a subunit the number of binding sites is reduced by one each time then we can model the process by utilising a site-exclusion heuristic. In this case the overall probability of binding, p, for the non-adjusted and adjusted dataset is

p=0.2450 and *p=0.2489* respectively. The predicted frequencies (rounded to integer) for one, two, three and four avidins being bound to a single TC is 1688, 304, 36, 2 and 1688, 315, 39 and 2 for the two respective cases. As seen in Fig. S4C this model also follows the overall trend of the data.

SUPPORTING TABLES, FIGURES AND LEGENDS

Supporting Table S1: Strains and plasmids used in this work

Strain name	Description (strain; plasmid[s])	Reference
**Background strains**		
WT	Wild-type M90T, serotype 5a	(Sansonetti *et al.*, 1982)
Δ*ipaB*	SF620	(Menard *et al.*, 1993)
Δ*ipaD*	SF622	(Menard *et al.*, 1993)
Δ*mxiG* Δ*ipaD*	Double mutant Δ*mxiG* Δ*ipaD*	This study[a]
Δ*mxiG* Δ*ipaB*	Double mutant Δ*mxiG* Δ*ipaB*	This study
Δ*mxiG* Δ*mxiH*	Double mutant Δ*mxiG* Δ*mxiH*	This study
Δ*ipaB* Δ*ipaD*	Double mutant Δ*ipaB* Δ*ipaD*	(Roehrich *et al.*, 2013)[a]
Δ*mxiH* Δ*ipaD*	Double mutant Δ*mxiH* Δ*ipaD*	This study[a]
$ipaD^{WT}$	SF622; pWPsf4D	(Picking *et al.*, 2005)
ipaD	SF622; pUC18 $ipaD_{C322S}$	This study
**Avidin-labeling strains**		
$ipaD_{avi}$	Δ*mxiG* Δ*ipaD*; pBAD::*mxiG* $ipaD_{avi}$	This study
$ipaB_{avi}$	Δ*mxiG* Δ*ipaB*; pBAD::*mxiG* $ipaB_{avi}$	This study
**WT and *ipaB*- NC purification strains and FACS**		
WT (for NCs) or $mxiG^{-/+}$ (for FACS)	Δ*mxiG* pSZ1 (pBAD::His_6*mxiG*)	(Zenk *et al.*, 2007)
ipaB-	Δ*mxiG* Δ*ipaB;* pSZ1	(Zenk *et al.*, 2007); this study
**MxiH mutant NC purification strains and FACS**		
$mxiH_{Q51A}$	Δ*mxiG* Δ*mxiH*; pSZ1, pACT3 $mxiH_{Q51A}$	(Zenk *et al.*, 2007); this study
$mxiH_{P44A}$	Δ*mxiG* Δ*mxiH*; pSZ1, pACT3 $mxiH_{P44A}$	(Zenk *et al.*, 2007); this study
$mxiH_{P44A/Q51A}$	Δ*mxiG* Δ*mxiH*; pSZ1, pACT3 $mxiH_{Q51A+P44A}$	(Zenk *et al.*, 2007); this study
**IpaD single cysteine mutants**		
$ipaD_{V170C}$	SF622; pUC18 $ipaD_{C322S/V170C}$	This study
$ipaD_{L171C}$	SF622; pUC18 $ipaD_{C322S/L171C}$	This study
$ipaD_{S172C}$	SF622; pUC18 $ipaD_{C322S/S172C}$	This study
$ipaD_{S173C}$	SF622; pUC18 $ipaD_{C322S/S173C}$	This study
$ipaD_{L174C}$	SF622; pUC18 $ipaD_{C322S/L174C}$	This study
$ipaD_{L257C}$	SF622; pUC18 $ipaD_{C322S/L257C}$	This study
$ipaD_{K258C}$	SF622; pUC18 $ipaD_{C322S/K258C}$	This study
$ipaD_{S259C}$	SF622; pUC18 $ipaD_{C322S/S259C}$	This study
$ipaD_{L260C}$	SF622; pUC18 $ipaD_{C322S/L260C}$	This study
$ipaD_{D261C}$	SF622; pUC18 $ipaD_{C322S/D261C}$	This study
**IpaD double cysteine mutants**		
$ipaD_{V170C/L257C}$	SF622; pUC18 $ipaD_{C322S/V170C/L257C}$	This study
$ipaD_{V170C/K258C}$	SF622; pUC18 $ipaD_{C322S/V170C/K258C}$	This study
$ipaD_{V170C/S259C}$	SF622; pUC18 $ipaD_{C322S/V170C/S259C}$	This study
$ipaD_{V170C/L260C}$	SF622; pUC18 $ipaD_{C322S/V170C/L260C}$	This study
$ipaD_{V170C/D261C}$	SF622; pUC18 $ipaD_{C322SV170C/D261C}$	This study
$ipaD_{L171C/L257C}$	SF622; pUC18 $ipaD_{C322S/L171C/L257C}$	This study
$ipaD_{L171C/K258C}$	SF622; pUC18 $ipaD_{C322S/L171C/K258C}$	This study
$ipaD_{L171C/S259C}$	SF622; pUC18 $ipaD_{C322S/L171C/S259C}$	This study
$ipaD_{L171C/L260C}$	SF622; pUC18 $ipaD_{C322S/L171C/L260C}$	This study
$ipaD_{L171C/D261C}$	SF622; pUC18 $ipaD_{C322S/L171C/D261C}$	This study
$ipaD_{S172C/L257C}$	SF622; pUC18 $ipaD_{C322S/S172C/L257C}$	This study
$ipaD_{S172C/K258C}$	SF622; pUC18 $ipaD_{C322S/S172C/K258C}$	This study
$ipaD_{S172C/S259C}$	SF622; pUC18 $ipaD_{C322S/S172C/S259C}$	This study
$ipaD_{S172C/L260C}$	SF622; pUC18 $ipaD_{C322S/S172C/L260C}$	This study

$ipaD_{S172C/D261C}$	SF622; pUC18 $ipaD_{C322S/S172C/D261C}$	This study
$ipaD_{S173C/L257C}$	SF622; pUC18 $ipaD_{C322S/S173C/L257C}$	This study
$ipaD_{S173C/K258C}$	SF622; pUC18 $ipaD_{C322S/S173C/K258C}$	This study
$ipaD_{S173C/S259C}$	SF622; pUC18 $ipaD_{C322S/S173C/S259C}$	This study
$ipaD_{S173C/L260C}$	SF622; pUC18 $ipaD_{C322S/S173C/L260C}$	This study
$ipaD_{S173C/D261C}$	SF622; pUC18 $ipaD_{C322S/S173C/D261C}$	This study
$ipaD_{L174C/L257C}$	SF622; pUC18 $ipaD_{C322S/L174C/L257C}$	This study
$ipaD_{L174C/K258C}$	SF622; pUC18 $ipaD_{C322S/L174C/K258C}$	This study
$ipaD_{L174C/S259C}$	SF622; pUC18 $ipaD_{C322S/L174C/S259C}$	This study
$ipaD_{L174C/L260C}$	SF622; pUC18 $ipaD_{C322S/L174C/L260C}$	This study
$ipaD_{L174C/D261C}$	SF622; pUC18 $ipaD_{C322S/L174C/D261C}$	This study
**IpaD cysteine mutants overexpressing *mxiH* for crosslinking via long needles**		
*WT	Wild-type M90T; pACT3 *mxiH*	(Shen *et al.*, 2012)
*$\Delta ipaD$	SF622; pACT3 *mxiH*	This study
*$ipaD$	SF622; pACT3 *mxiH*, pUC18 $ipaD_{C322S}$	This study
*$ipaD_{V170C}$	SF622; pACT3 *mxiH*, pUC18 $ipaD_{C322S/V170C}$	This study
*$ipaD_{L171C}$	SF622; pACT3 *mxiH*, pUC18 $ipaD_{C322S/L171C}$	This study
*$ipaD_{S172C}$	SF622; pACT3 *mxiH*, pUC18 $ipaD_{C322S/S172C}$	This study
*$ipaD_{S173C}$	SF622; pACT3 *mxiH*, pUC18 $ipaD_{C322S/S173C}$	This study
*$ipaD_{L174C}$	SF622; pACT3 *mxiH*, pUC18 $ipaD_{C322S/L174C}$	This study
*$ipaD_{L257C}$	SF622; pACT3 *mxiH*, pUC18 $ipaD_{C322S/L257C}$	This study
*$ipaD_{K258C}$	SF622; pACT3 *mxiH*, pUC18 $ipaD_{C322S/K258C}$	This study
*$ipaD_{S259C}$	SF622; pACT3 *mxiH*, pUC18 $ipaD_{C322S/S259C}$	This study
*$ipaD_{L260C}$	SF622; pACT3 *mxiH*, pUC18 $ipaD_{C322S/L260C}$	This study
*$ipaD_{D261C}$	SF622; pACT3 *mxiH*, pUC18 $ipaD_{C322S/D261C}$	This study
*$ipaD_{V170C/L257C}$	SF622; pACT3 *mxiH*, pUC18 $ipaD_{C322S/V170C/L257C}$	This study
*$ipaD_{V170C/K258C}$	SF622; pACT3 *mxiH*, pUC18 $ipaD_{C322S/V170C/K258C}$	This study
*$ipaD_{V170C/S259C}$	SF622; pACT3 *mxiH*, pUC18 $ipaD_{C322S/V170C/S259C}$	This study
*$ipaD_{V170C/L260C}$	SF622; pACT3 *mxiH*, pUC18 $ipaD_{C322S/V170C/L260C}$	This study
*$ipaD_{V170C/D261C}$	SF622; pACT3 *mxiH*, pUC18 $ipaD_{C322SV170C/D261C}$	This study
*$ipaD_{L171C/L257C}$	SF622; pACT3 *mxiH*, pUC18 $ipaD_{C322S/L171C/L257C}$	This study
*$ipaD_{L171C/K258C}$	SF622; pACT3 *mxiH*, pUC18 $ipaD_{C322S/L171C/K258C}$	This study
*$ipaD_{L171C/S259C}$	SF622; pACT3 *mxiH*, pUC18 $ipaD_{C322S/L171C/S259C}$	This study
*$ipaD_{L171C/L260C}$	SF622; pACT3 *mxiH*, pUC18 $ipaD_{C322S/L171C/L260C}$	This study
*$ipaD_{L171C/D261C}$	SF622; pACT3 *mxiH*, pUC18 $ipaD_{C322S/L171C/D261C}$	This study
*$ipaD_{S172C/L257C}$	SF622; pACT3 *mxiH*, pUC18 $ipaD_{C322S/S172C/L257C}$	This study
*$ipaD_{S172C/K258C}$	SF622; pACT3 *mxiH*, pUC18 $ipaD_{C322S/S172C/K258C}$	This study
*$ipaD_{S172C/S259C}$	SF622; pACT3 *mxiH*, pUC18 $ipaD_{C322S/S172C/S259C}$	This study
*$ipaD_{S172C/L260C}$	SF622; pACT3 *mxiH*, pUC18 $ipaD_{C322S/S172C/L260C}$	This study
*$ipaD_{S172C/D261C}$	SF622; pACT3 *mxiH*, pUC18 $ipaD_{C322S/S172C/L261C}$	This study
*$ipaD_{S173C/L257C}$	SF622; pACT3 *mxiH*, pUC18 $ipaD_{C322S/S173C/L257C}$	This study
*$ipaD_{S173C/K258C}$	SF622; pACT3 *mxiH*, pUC18 $ipaD_{C322S/S173C/K258C}$	This study
*$ipaD_{S173C/S259C}$	SF622; pACT3 *mxiH*, pUC18 $ipaD_{C322S/S173C/S259C}$	This study
*$ipaD_{S173C/L260C}$	SF622; pACT3 *mxiH*, pUC18 $ipaD_{C322S/S173C/L260C}$	This study
*$ipaD_{S173C/D261C}$	SF622; pACT3 *mxiH*, pUC18 $ipaD_{C322S/S173C/L261C}$	This study
*$ipaD_{L174C/L257C}$	SF622; pACT3 *mxiH*, pUC18 $ipaD_{C322S/L174C/L257C}$	This study
*$ipaD_{L174C/K258C}$	SF622; pACT3 *mxiH*, pUC18 $ipaD_{C322S/L174C/K258C}$	This study
*$ipaD_{L174C/S259C}$	SF622; pACT3 *mxiH*, pUC18 $ipaD_{C322S/L174C/S259C}$	This study
*$ipaD_{L174C/L260C}$	SF622; pACT3 *mxiH*, pUC18 $ipaD_{C322S/L174C/L260C}$	This study
*$ipaD_{L174C/D261C}$	SF622; pACT3 *mxiH*, pUC18 $ipaD_{C322S/L174C/D261C}$	This study
**IpaD cysteine mutants for crosslinking in crude needle complex preparations**		
$\Delta mxiH$ $\Delta ipaD$; *ipaD*	$\Delta mxiH$ $\Delta ipaD$; pUC18 $ipaD_{C322S}$	This study
$\Delta mxiH$ $\Delta ipaD$; $ipaD_{S173C}$	$\Delta mxiH$ $\Delta ipaD$; pUC18 $ipaD_{C322S/S173C}$	This study
$\Delta mxiH$ $\Delta ipaD$; $ipaD_{K258C}$	$\Delta mxiH$ $\Delta ipaD$; pUC18 $ipaD_{C322S/K258C}$	This study
$\Delta mxiH$ $\Delta ipaD$; $ipaD_{S173C/258C}$	$\Delta mxiH$ $\Delta ipaD$; pUC18 $ipaD_{C322S/S173C/K258C}$	This study
$\Delta mxiH$ $\Delta ipaD$; *mxiH ipaD*	$\Delta mxiH$ $\Delta ipaD$; pACT3 *mxiH*, pUC18_O^c_ $ipaD_{C322S}$	This study
$\Delta mxiH$ $\Delta ipaD$; *mxiH* $ipaD_{S173C}$	$\Delta mxiH$ $\Delta ipaD$; pACT3 *mxiH*, pUC18_O^c_ $ipaD_{C322S/S173C}$	This study
$\Delta mxiH$ $\Delta ipaD$; *mxiH* $ipaD_{K258C}$	$\Delta mxiH$ $\Delta ipaD$; pACT3 *mxiH*, pUC18_O^c_ $ipaD_{C322S/K258C}$	This study

Δ*mxiH* Δ*ipaD*; *mxiH* $ipaD_{S173C/K258C}$	Δ*mxiH* Δ*ipaD*; pACT3 *mxiH*, pUC18_O^c_ $ipaD_{C322S/S173C/K258C}$	This study
Δ*ipaB* Δ*ipaD*; *ipaD*	Δ*ipaB* Δ*ipaD*; pUC18 $ipaD_{C322S}$	This study
Δ*ipaB* Δ*ipaD*; $ipaD_{S173C}$	Δ*ipaB* Δ*ipaD*; pUC18 $ipaD_{C322S/S173C}$	This study
Δ*ipaB* Δ*ipaD*; $ipaD_{K258C}$	Δ*ipaB* Δ*ipaD*; pUC18 $ipaD_{C322S/K258C}$	This study
Δ*ipaB* Δ*ipaD*; $ipaD_{S173C/K258C}$	Δ*ipaB* Δ*ipaD*; pUC18 $ipaD_{C322S/S173C/K258C}$	This study
Δ*mxiH* Δ*ipaD*; $mxiH_{Q51A}$ *ipaD*	Δ*mxiH* Δ*ipaD*; pACT3 $mxiH_{Q51A}$, pUC18_O^c_$ipaD_{C322S}$	This study
Δ*mxiH* Δ*ipaD*; $mxiH_{Q51A}$ $ipaD_{S173C}$	Δ*mxiH* Δ*ipaD*; pACT3 $mxiH_{Q51A}$, pUC18_O^c_$ipaD_{C322S/S173C}$	This study
Δ*mxiH* Δ*ipaD*; $mxiH_{Q51A}$ $ipaD_{K258C}$	Δ*mxiH* Δ*ipaD*; pACT3 $mxiH_{Q51A}$, pUC18_O^c_$ipaD_{C322S/K258C}$	This study
Δ*mxiH* Δ*ipaD*; $mxiH_{Q51A}$ $ipaD_{S173C/258C}$	Δ*mxiH* Δ*ipaD*; pACT3 $mxiH_{Q51A}$, pUC18_O^c_$ipaD_{C322S/S173C/K258C}$	This study

[a]We have noticed that all strains made with these backgrounds express less, and hence secrete little IpaA. We believe that this is due to the manner in which *ipaD,* which lies directly upstream of *ipaA,* was inactivated in them. However, this has no bearing on the study described here.

Supporting Table S2. Primers used in this study

primers	Sequence
**For double knockout background strains**	
IpaD_KO_tetF	AATAATATATGGCTCTTCCTGTAAGGAAATAACCATGAATATAACAACTTA AGACCCACTTTCA
IpaD_KO_tetR	TATTATTTACATTATGCATGGCGCACCTCAGAAATGGAGAAACATATGAA TATCCTCCTTACTAAGCACTTGTCTCCTG
IpaB_KO_tetF	CAATCATACTTGGACGCAATTCAGGATATCAAGGAGTAATTATTATGCAT AATGTAAGTTAAGACCCACTTTCA
IpaB_KO_tetR	GTGTTTTGAATTTCCATAACATTCTCCTTATTTGTATCAAGCAGTAGTTTG CATATGAATATCCTCCTTACTAAGCACTTGTCTCCTG
MxiGH_KO_F1	GAGAGCAAGCAGAATAATCGAAGGATATAAGAGGATTTATGTCTGGTGT AGGCTGGAGCTGCTTC
MxiH_KO_R1	GGATAAATGTAATTCATGAATGCTCCCTATTATCTGAAGTTTTGAATAATC ATATGAATATCCTCCTTAG
**For introduction of *mxiH* point mutations into pACT3**	
mxiHP44A_for	TCCTTCGAATGCACAGTTGCTGGCTGAATAC
mxiHP44A_rev	GTATTCAGCCAGCAACTGTGCATTCGAAGG
mxiHQ51A_for	CGAATCCACAGGCGCTGGCTGAATACCAAAG
mxiHQ51A_rev	CTTTGGTATTCAGCCAGCGCCTGTGGATTCGAAG
mxiHP44Q51A_for	TGCTGGCTGAATACGCAATAAATTATCTG
mxiHP44Q51A_for	CAGATAATTTACTTGCGTATTCCAGCCAGCAAC
**For *ipaD*$_{avi}$ and *ipaB*$_{avi}$ strains**	
ipaD_NdeI_F1	GATTACGAATTCCATATGAATATAACAACTC
paD_EcoRV_R1	CTCTATGAGATATCATTTGATCTCC
avitagD_F1	GGTCTGAACGACATATTCGAAGCTCAGAAAATGAATGGCACGAACTGGG TACGAGGGGTTCTCCTGTAAGTTCCTT ACTATGC
avitagD_R1	TTCGTGCCATTCGATTTTCTGAGCTTCGAATATGTCGTTCAGACCACTTC CACGCGTGCCCAAATGAGAACTGGT CGTTGTTTT
ipaD_Xma_F	GTGAGGTACCCGGGCTCTTCCTGTAAGGAAATAACCATGAATATAAC
ipaD_Cla_R	GGCAGGTACCATCGATTCAGAAATGGAGAAAAAGTTTATC
ipaB_HindIII_F1	GGGGAAGCTTGATGCATAATGTAAGCACCACAAC
ipaB_PstI_R1	GGGGCTGCAGTCCTTATTTGTATCAAGCAGTAGT
avitagB_F1	GGTCTGAACGACATATTCGAAGCTCAGAAAATCGAATGGCACGAACTGG GTACGAGGGGTTCTGCTAACAAATTATTTTCTCTT ACAATTG
avitagB_R1	TTCGTGCCATTCGATTTTCTGAGCTTCGAATATGTCGTTCAGACCACTTC CACGCGTGCCCAATGCATCATTTGCAGCTTGGATAG
ipaB_Xma_F	GTGAGGTA*CCCGGG*ATATCAAGGAGTAATTATTATGCATAATG
ipaB_Cla_R	GGCAGGTACCATCGATTCAAGCAGTAGTTTGTTGCAAAATTG
**For *ipaD* mutations introduced via one-step PCR or two-step PCR**	
ipaD_EcoRV_for	GGAGATCAAATGATATCTCATAG
ipaD_PstI_rev	CTAGCTGCAGTCAGAAATGGAGAAAAAG
**For background *ipaD*$_{C322S}$ (one-step PCR, used with ipaD_EcoRV_for)**	
ipaD_C322S_rev	CTAGCTGCAGTCAGAAATGGAGAAAAAGTTTATCTGTATCTGT*AgA*TGAG CTTATTG
**For *ipaD* cysteine mutants (two-step PCR)**	
ipaD_V170C_for	GTATCAAGATTTTAGCGCT*tgT*CTTTCCAGTCTTGCC
ipaD_V170C_rev	GGCAAGACTGGAAAG*Aca*AGCGCTAAAATCTTGATAC
ipaD_L171C_for	GTATCAAGATTTTAGCGCTGTT*tgT*TCCAGTCTTGCC
ipaD_L171C_rev	GGCAAGACTGGA*Aca*AACAGCGCTAAAATCTTGATAC
ipaD_S172C_for	GATTTTAGCGCTGTTCTT*TgC*AGTCTTGCCGGCTGGATCTC
ipaD_S172C_rev	GAGATCCAGCCGGCAAGACT*GcA*AAGAACAGCGCTAAAATC
ipaD_S173C_for	GATTTTAGCGCTGTTCTTTCC*tGT*CTTGCCGGCTGGATCTC
ipaD_S173C_rev	GAGATCCAGCCGGCAAG*ACa*GGAAAGAACAGCGCTAAAATC
ipaD_L174C_for	GATTTTAGCGCTGTTCTTTCCAGT*tgT*GCCGGCTGGATCTC
ipaD_L174C_rev	GAGATCCAGCCGGC*Aca*ACTGGAAAGAACAGCGCTAAAATC

ipaD_L257C_for	GACCCCAATAGACAATATG*Tgc*AAAAGCTTAGATAATCTAGG
ipaD_L257C_rev	CCTAGATTATCTAAGCTTTT*gcA*CATATTGTCTATTGGGGTC
ipaD_K258C_for	GACCCCAATAGACAATATGTTA*tgc*AGCTTAGATAATCTAGG
ipaD_K258C_rev	CCTAGATTATCTAAGCT*gca*TAACATATTGTCTATTGGGGTC
ipaD_S259C_for	GACCCCAATAGACAATATGTTAAAA*tGC*TTAGATAATCTAGG
ipaD_S259C_rev	CCTAGATTATCTAA*GCa*TTTTAACATATTGTCTATTGGGGTC
ipaD_L260C_for	GACAATATGTTAAAAAGC*Tgc*GATAATCTAGGTGGAAATG
ipaD_L260C_rev	CATTTCCACCTAGATTATC*gcA*GCTTTTTAACATATTGTC
ipaD_D261C_for	GACAATATGTTAAAAAGCTTA*tgT*AATCTAGGTGGAAATG
ipaD_D261C_rev	CATTTCCACCTAGATT*Aca*TAAGCTTTTTAACATATTGTC

*Underlined capital letters represent restriction endonuclease sites generated to facilitate cloning. Italic small letters correspond to point mutations.

Supporting Table S3. Crosslinking results for *ipaD* cysteine mutants

Strains designation	-	Oxidiser: Sodium tetrathionate	crosslinker: $BM(PEG)_2$
*$ipaD_{V170C}$	-	-	-
*$ipaD_{L171C}$	-	-	-
*$ipaD_{S172C}$	-	-	-
*$ipaD_{S173C}$	-	-	-
*$ipaD_{L174C}$	-	-	-
*$ipaD_{L257C}$	-	-	-
*$ipaD_{K258C}$	-	-	-
*$ipaD_{S259C}$	-	-	-
*$ipaD_{L260C}$	-	-	-
*$ipaD_{D261C}$	-	-	-
*$ipaD_{V170C/L257C}$	-	-	-
*$ipaD_{V170C/K258C}$	-	-	++[a]
*$ipaD_{V170C/S259C}$	-	-	-
*$ipaD_{V170C/L260C}$	-	-	-
*$ipaD_{V170C/D261C}$	-	-	+
*$ipaD_{L171C/L257C}$	-	-	-
*$ipaD_{L171C/K258C}$	-	-	-
*$ipaD_{L171C/S259C}$	-	-	-
*$ipaD_{L171C/L260C}$	-	-	-
*$ipaD_{L171C/D261C}$	-	-	-
*$ipaD_{S172C/L257C}$	-	-	-
*$ipaD_{S172C/K258C}$	-	-	++[a]
*$ipaD_{S172C/S259C}$	-	-	-/+
*$ipaD_{S172C/L260C}$	-	-	-/+
*$ipaD_{S172C/D261C}$	-	-	++[a]
*$ipaD_{S173C/L257C}$	-	-	-
*$ipaD_{S173C/K258C}$	-/+	-/+	++[a]
*$ipaD_{S173C/S259C}$	-	-	++[a]
*$ipaD_{S173C/L260C}$	-	-	-
*$ipaD_{S173C/D261C}$	-	-	+[a]
*$ipaD_{L174C/L257C}$	-	-	-
*$ipaD_{L174C/K258C}$	-	-	+[a]
*$ipaD_{L174C/S259C}$	-	-	-/+
*$ipaD_{L174C/L260C}$	-	-	-
*$ipaD_{L174C/D261C}$	-	-	++[a]

These data show the result of the initial screen of all mutants using the long needle shearing plus TCA precipitation method. The data shown are based on at least 2 independent assays giving similar results. Any high molecular weight band observed at ~100 kDa is succinctly described as strong (++), obvious (+), faint (-/+) and negative (-). [a]indicates strains where a second higher molecular weight band migrating at ~170 kDa was also seen. *corresponds to addition of pACT3*mxiH*, which allows MxiH overexpression and generation of long needles.

Table S4: Processing information for 3D reconstructions of TCs

	WT	Δ*ipaB*	$mxiH_{P44A}$	$mxiH_{Q51A}$	$mxiH_{P44A+Q51A}$
Number of micrographs	205	152	130	125	206
Total number of images in initial selection	2945	4225	2817	2523	2131
Total number of images in final map	2718	2573	2968	2545	2126
Number of class averages used	181	128	148	127	106
Approximate resolution (Å, FSC = 0.5)	24	25	23	21	24

REFERENCES

Bueler, S.A. & J.L. Rubinstein (2008) Location of subunit d in the peripheral stalk of the ATP synthase from *Saccharomyces cerevisiae*. *Biochemistry* **47**: 11804-11810.

Menard, R., P.J. Sansonetti & C. Parsot (1993) Non-polar mutagenesis of the ipa genes defines ipa B, Ipa C and IpaD as effectors of *Shigella* entry into epithelial cells. *J Bacteriol.* **175**: 5899-5906.

Picking, W.L., H. Nishioka, P.D. Hearn, M.A. Baxter, A.T. Harrington, A. Blocker & W.D. Picking (2005) IpaD of *Shigella flexneri* is independently required for regulation of Ipa protein secretion and efficient insertion of IpaB and IpaC into host membranes. *Infect Immun* **73**: 1432-1440.

Roehrich, A.D., E. Guillossou, A.J. Blocker & I. Martinez-Argudo (2013) *Shigella* IpaD has a dual role: signal transduction from the type III secretion system needle tip and intracellular secretion regulation. *Mol Microbiol* **87**: 690-706.

Roehrich, A.D., I. Martinez-Argudo, S. Johnson, A.J. Blocker & A.K. Veenendaal (2010) The extreme C terminus of *Shigella flexneri* IpaB is required for regulation of type III secretion, needle tip composition, and binding. *Infect Immun* **78**: 1682-1691.

Sansonetti, P.J., D.J. Kopecko & S.B. Formal (1982) Involvement of a plasmid in the invasive ability of *Shigella flexneri*. *Infect Immun* **35**: 852-860.

Shen, D.K., N. Moriya, I. Martinez-Argudo & A.J. Blocker (2012) Needle length control and the secretion substrate specificity switch are only loosely coupled in the type III secretion apparatus of *Shigella*. *Microbiology* **158**: 1884-1896.

Veenendaal, A.K., J.L. Hodgkinson, L. Schwarzer, D. Stabat, S.F. Zenk & A.J. Blocker (2007) The type III secretion system needle tip complex mediates host cell sensing and translocon insertion. *Mol Microbiol* **63**: 1719-1730.

Wilson, K.P., L.M. Shewchuk, R.G. Brennan, A.J. Otsuka & B.W. Matthews (1992) *Escherichia coli* biotin holoenzyme synthetase/bio repressor crystal structure delineates the biotin- and DNA-binding domains. *Proc Natl Acad Sci U S A* **89**: 9257-9261.

Zenk, S.F., D. Stabat, J.L. Hodgkinson, A.K. Veenendaal, S. Johnson & A.J. Blocker (2007) Identification of minor inner-membrane components of the *Shigella* type III secretion system 'needle complex'. *Microbiology* **153**: 2405-2415.

Figure S1. Validation of cysteine-mediated crosslinking strains and assays. Single cysteine *ipaD* mutants **(A)** and double *ipaD* cysteine mutants **(B)** were checked for the level of *ipaD* expression by immunoblotting with anti-IpaD antibodies (*top*), their Ipa protein leakage was assessed using silver-staining of culture supernatants after SDS-PAGE (*middle*) and their capacity to cause contact hemolysis of sheep red blood cells also examined (*bottom*). **(C)** IpaD detection from crudely purified needle samples. Crude needles prepared from exponentially grown WT or *WT (WT overexpressing MxiH) were checked by immunoblotting with anti-IpaD and anti-MxiH as indicated. Only when long needles are generated is a strong monomeric IpaD signal seen. 1 and 3, samples equivalent to 1 or 3 ml culture of OD_{600} 1, respectively. **(D)** Crosslinking of specific IpaD cysteine mutants overexpressing *mxiH* in the presence of CR. Crude needles were either treated (+) or not (-) with Congo red (CR) before the addition (+) or not (-) of $BM(PEG)_2$ (X), and immunoblotted with anti-IpaD antibodies. The main high molecular weight band is indicated by an arrow. For this figure only, TCA precipitation was replaced by NaCl and PEG6000 mediated precipitation (Cordes *et al.*, 2005).

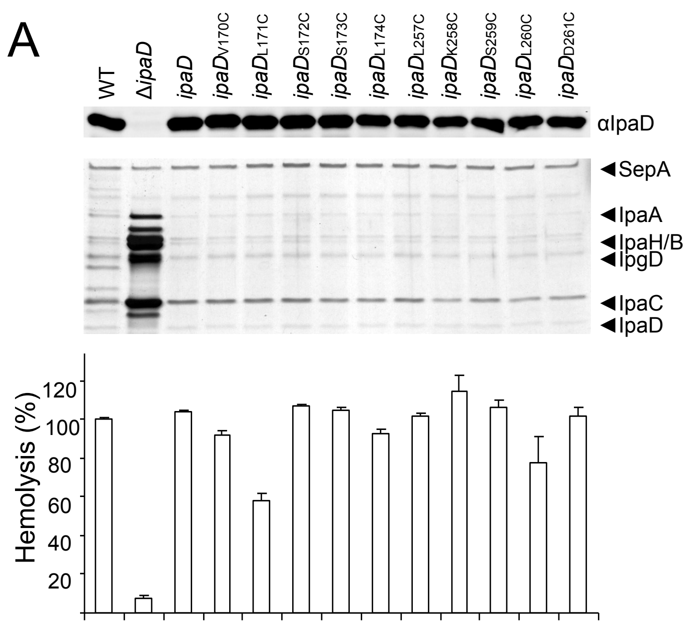

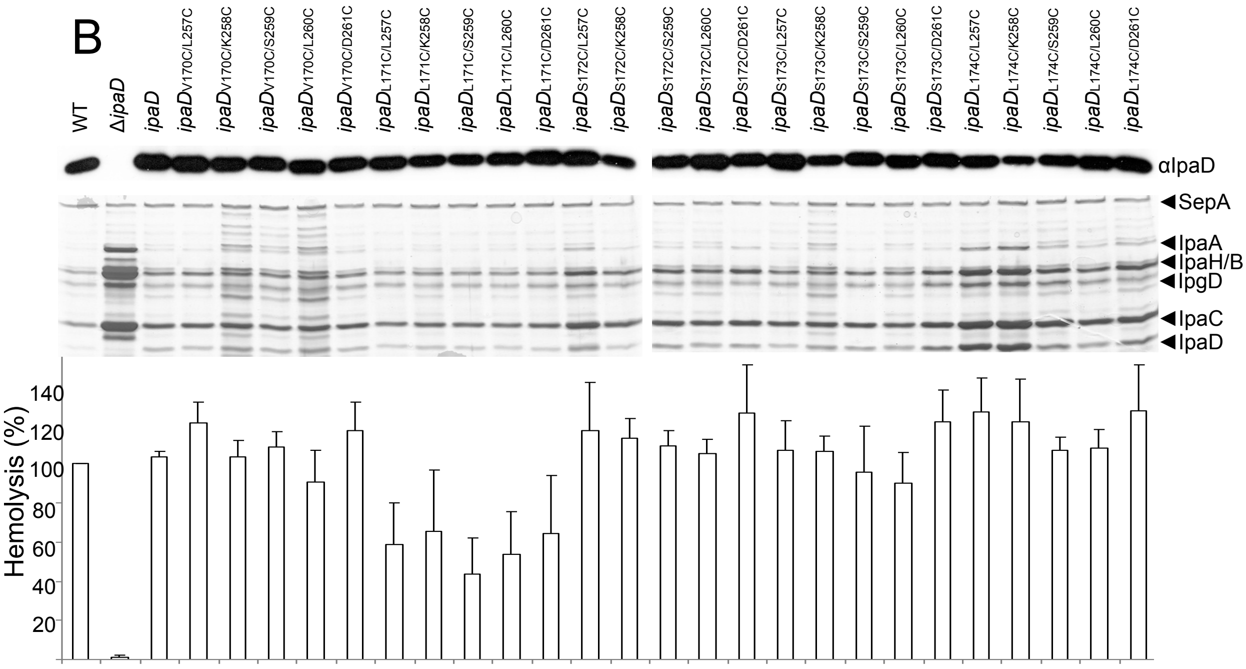

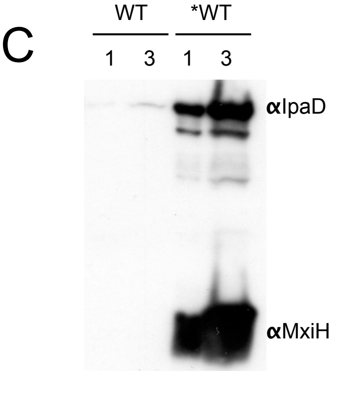

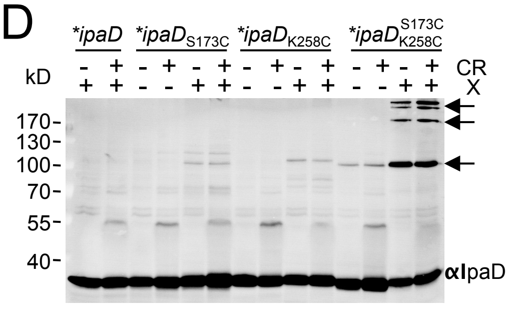

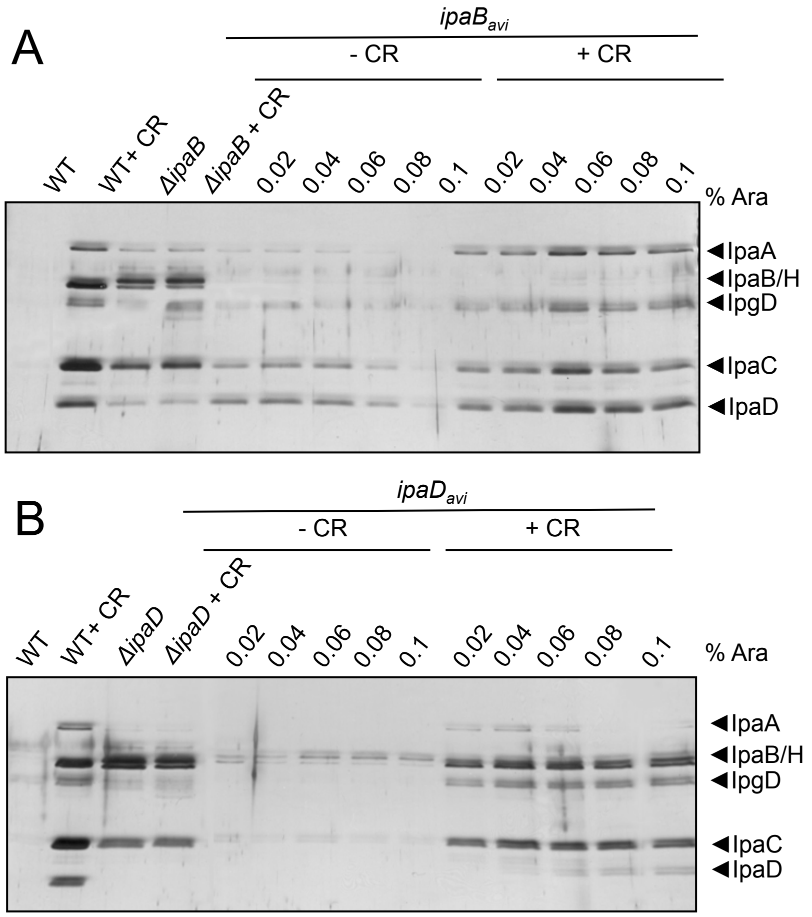

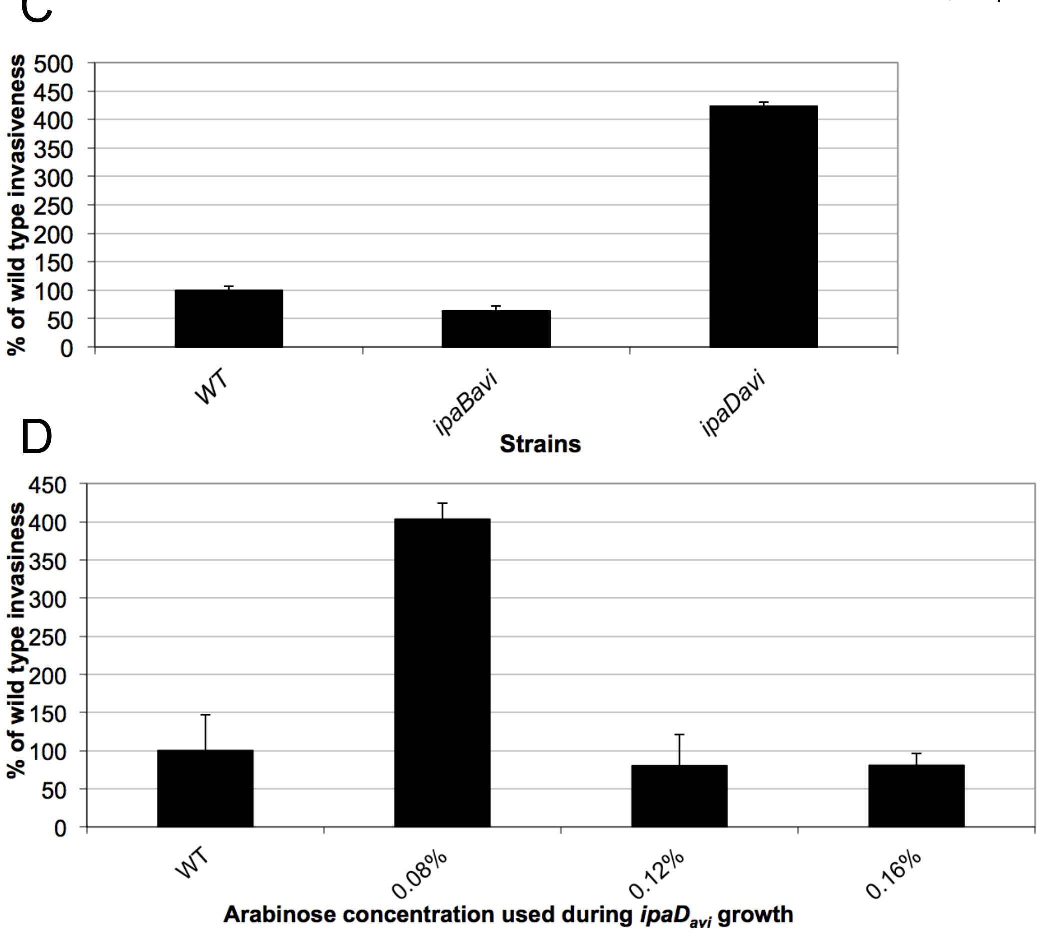

Figure S2. Validation of arabinose concentration used for growth to mid-exponential phase of *ipaB$_{avi}$* and *ipaD$_{avi}$* strains, by CR induction of Ipa proteins. Bacterial strains expressing IpaB_avitag **(A)** and IpaD_avitag **(B)** were grown to mid-exponential phase, in the presence of increasing concentrations of arabinose. TC composition was assessed by the secretion phenotype in response to the artificial inducer molecule CR (silver stained 10% SDS-PA gel). **HeLa cell invasion assay to determine TC function of *ipaB$_{avi}$* and *ipaD$_{avi}$* strains.** **(C)** Strains with IpaB_avitag and IpaD_avitag were grown to mid-exponential phase in the presence of 0.08% arabinose and assessed for their ability to invade HeLa cells. *ipaB$_{avi}$* and *ipaD$_{avi}$* strains had invasion efficiencies 64% ± 8.5 and 422% ± 70.9 of wild type levels, respectively. **(D)** *ipaD$_{avi}$* was grown to mid-exponential phase in the presence of increasing concentrations of arabinose and then assessed for its ability to invade HeLa cells. The invasion efficiencies for 0.08, 0.12 and 0.16% arabinose were 403% ±20.7, 80.2% ±21.3 and 80.6% ±15.3 of wild type levels, respectively.

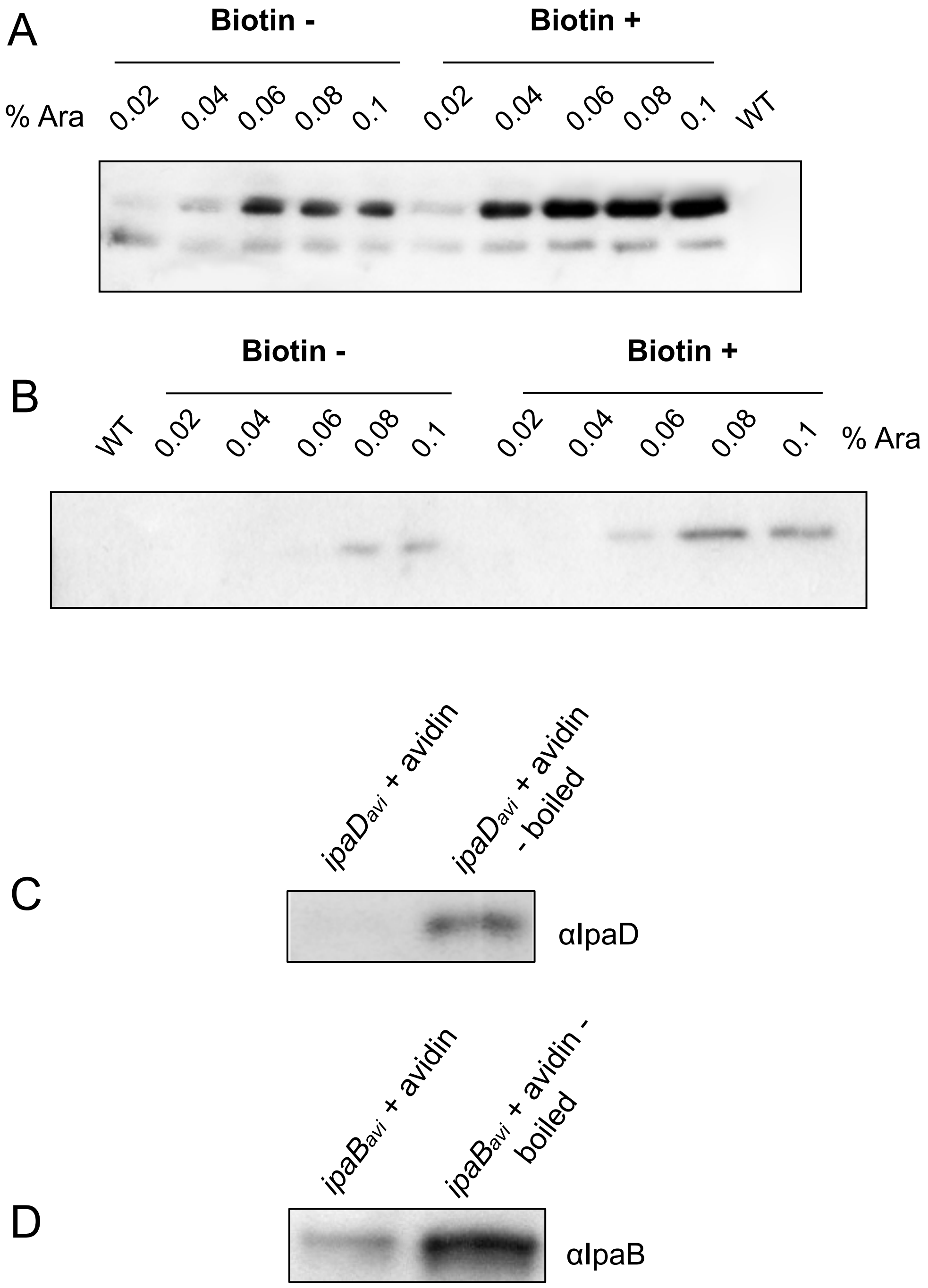

Figure S3. **IpaB_avitag and IpaD_avitag biotinylation when *ipaB*$_{avi}$ and *ipaD*$_{avi}$ are grown in the presence of biotin.** Bacterial cultures were grown to mid-exponential phase with increasing concentrations of arabinose (0.02 to 0.1%) and either with or without the addition of 0.05 mM biotin. The degree of biotinylation was assessed by Western blot with a Streptavidin-HRP conjugate. **(A)** IpaB_avitag showed significant biotinlyation without exogenous biotin, but was enhanced when grown with exogenous biotin. **(B)** IpaD_avitag showed only weak levels of biotinylation in the absence of exogenous biotin, but also showed enhancement of biotinlyation when grown with biotin. **Shift assay to establish avidin binding efficiency of *ipaB*$_{avi}$ and *ipaD*$_{avi}$ strains.** The principle of this assay is described in the Supplementary Information, under *Optimisation of avidin binding*. To establish the avidin labelling efficiency of NCs purified from *ipaB*$_{avi}$ and *ipaD*$_{avi}$ strains, purified labelled NCs were mixed with Laemmli sample buffer and run on an SDS-PA gel (10%) either with or without boiling of the sample. Detection of **(C)** IpaD and **(D)** IpaB revealed labelling efficiencies of approximately 100% and 80% respectively.

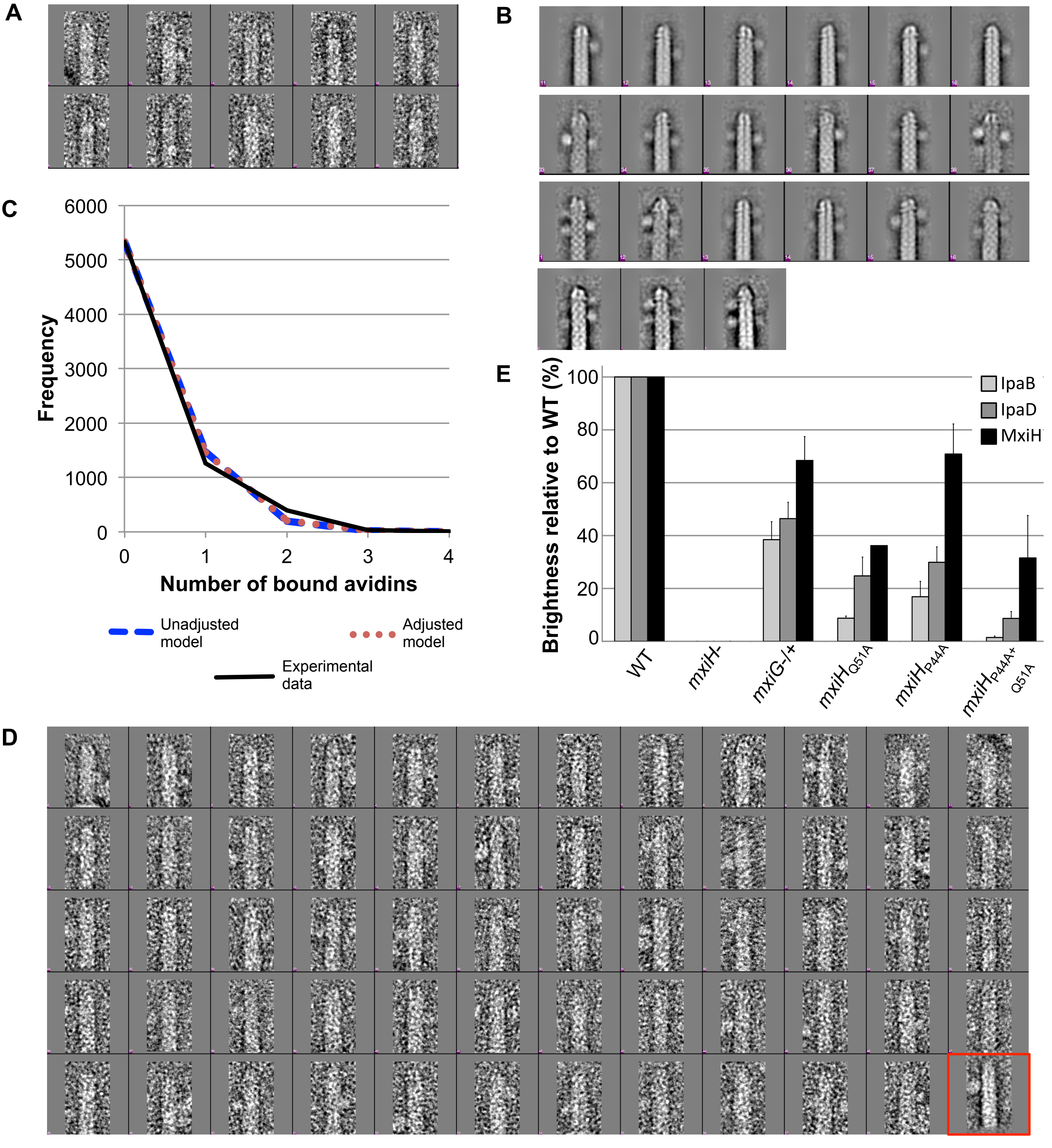

Figure S4: Avidin labelling of TC subunits and analysis of presence of IpaB, IpaD and MxiH on the surface of WT and *mxiH* mutants by flow cytometry. (A) Representative raw images of avidin bound to TCs of isolated NCs of the $ipaD_{avi}$ strain. **(B)** Representative images of the $ipaD_{avi}$ dataset reconstituted using the first 25 factors after Eigen-decomposition by correspondence analysis. Images with 1, 2, 3 and 4 bound avidin molecules are displayed (from top to bottom). **(C)** The number of images with either 1, 2, 3 or 4 bound avidin molecules is in good agreement with the predicted frequencies if avidin binding is modelled as a Poisson process (*unadjusted model* including ambiguous images, λ=0.2750; *adjusted model* excluding ambiguous images, λ=0.2862, as explained in the Supplementary Information). **(D)** 57 raw images from the $ipaB_{avi}$ dataset judged to contain avidin bound to the TC; *boxed in red* is the result of the classification of the 57 images using refine2D (EMAN) which produced only one class of 8 images where bound avidin could be seen in the class average. **(E)** The mutants listed were analysed by FACS using antibodies against MxiH, IpaD and IpaB. Data were normalised for the WT strain after subtraction of background. The data presented are the means of the geometric means from two experiments. The standard deviations of the means are indicated with bars.

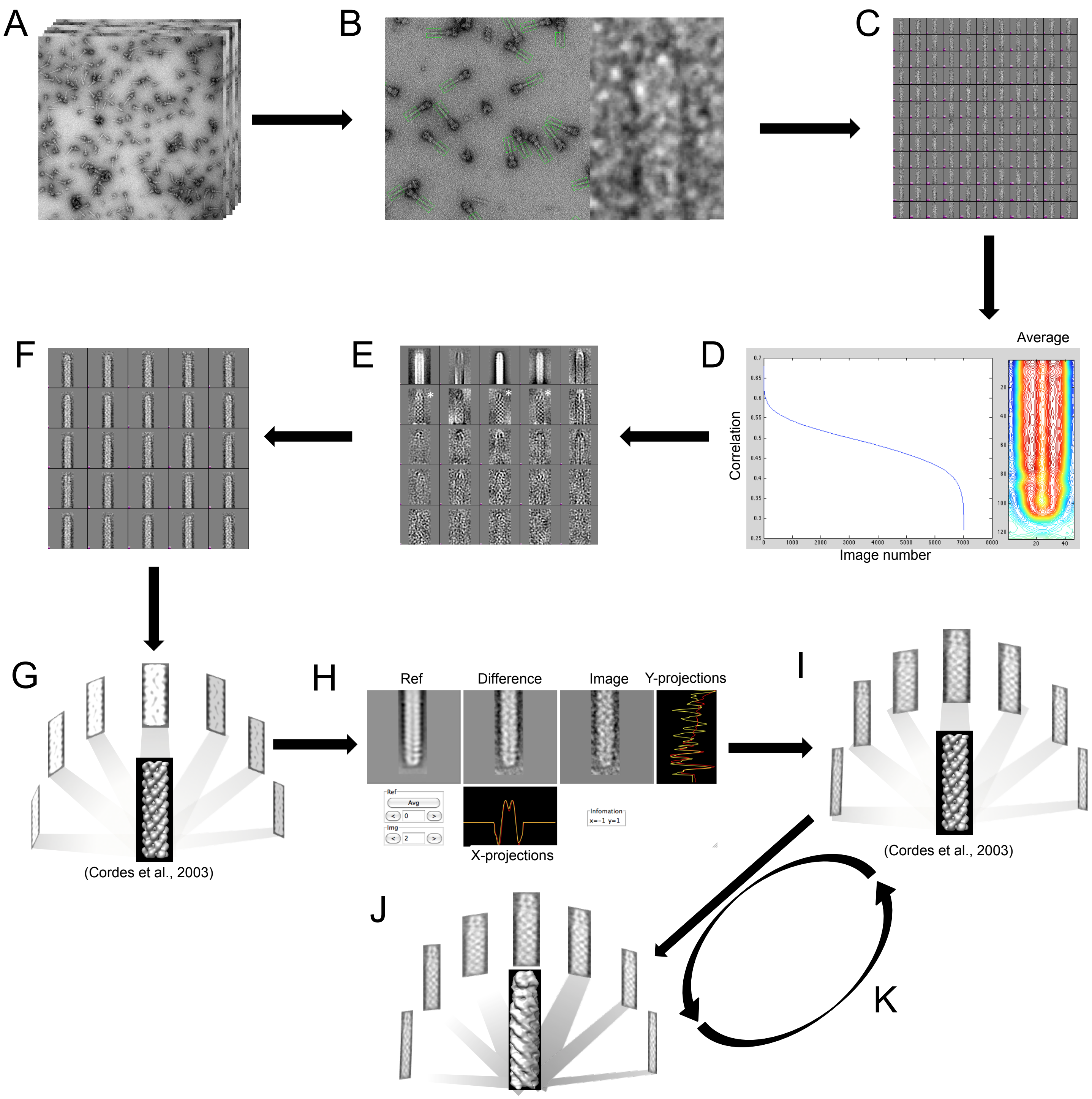

Figure S5. 3D reconstruction scheme developed for the TC. (A) Negative stain EM images were collected (50,000x magnification). **(B)** TCs were selected from individual NCs using helixboxer, part of the EMAN2 software package (*left*, boxing program; *right*, magnified single particle). **(C)** The selected images were merged into a single dataset file. **(D)** X,Y- shifts of each image were corrected by alignment with an average of all the images (*right*). The cross-correlations of each image and the average were calculated and sorted depending on the degree of correlation (*left*). Images with a correlation below 0.3 were discarded. **(E)** Eigen-decomposition by correspondence analysis produced eigenimages, displaying the inter-image variance. The eigenimages displaying the strongest helical patterns (*) were used to classify the images and class averages were formed **(F)**. **(G)** Class averages were aligned to projections of the 16 Å 3D map of the needle (Cordes et al., 2003) to correct for X,Y-shifts and rotations. **(H)** Y,X-shifts were further refined using a bespoke alignment program, where classes could be manually shifted against X and Y projections of a reference image (which was the sum of all the images) by visually inspecting the difference between the image and reference. **(I)** The azimuthal orientations of the class averages were determined by projection matching to the 16 Å needle, and the 3D map produced by back projecting the class averages into a single volume **(J)**. **(K)** The map was refined by imposing helical symmetry (only in the needle portion of the map) to create a new reference, and class averages were realigned to the new reference. This was iterated five times.

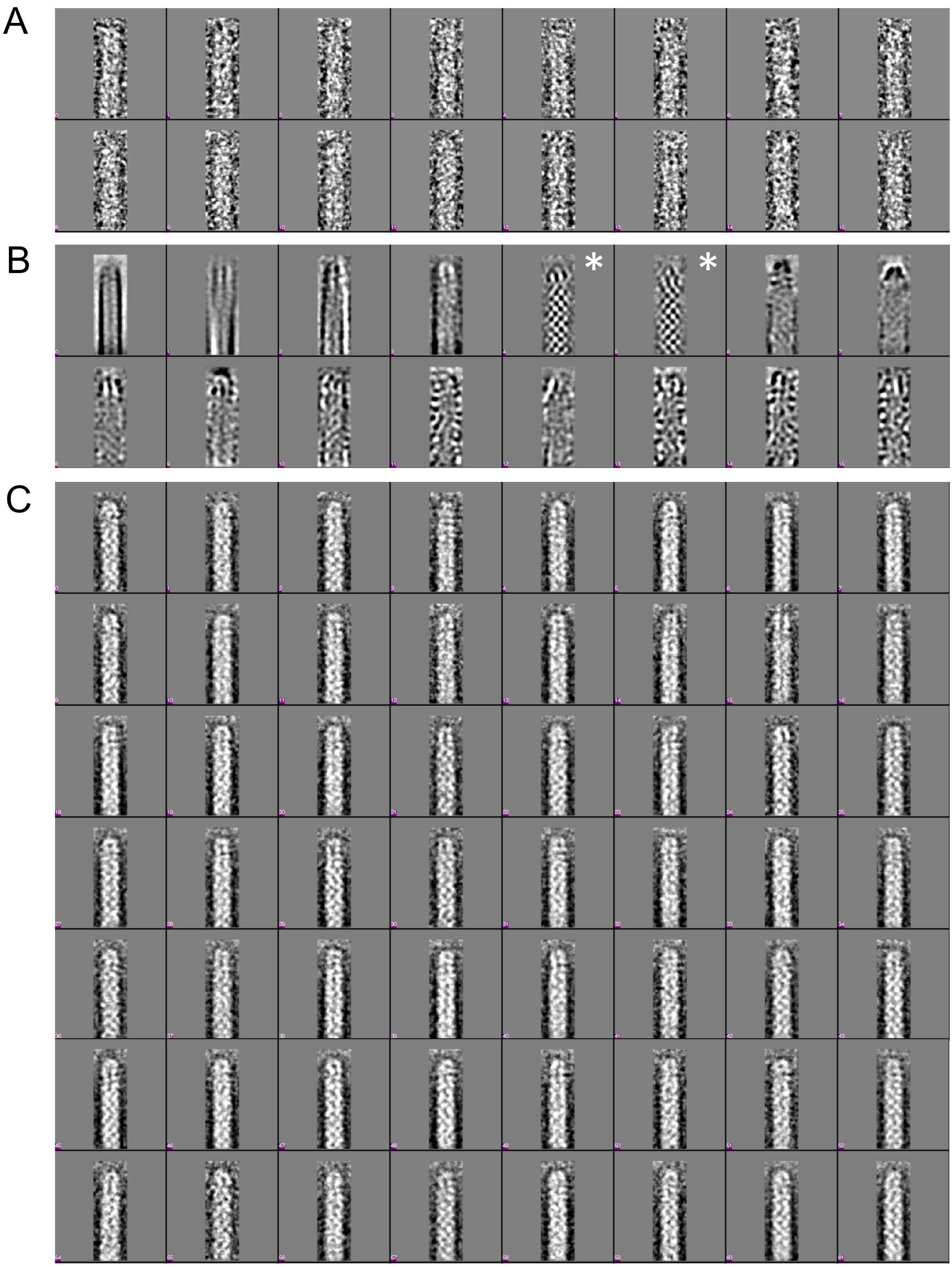

Figure S6. Image analysis of WT images from negative stain EM micrographs. (A) Representative images of singe particle images. **(B)** First 16 eigenimages produced by eigen-decomposition by correspondence analysis of the dataset; (*) eigenimages displaying strong helical information. **(C)** Class averages produced by classification of the dataset, using only the eigenimages/factors containing strong helical information.

A

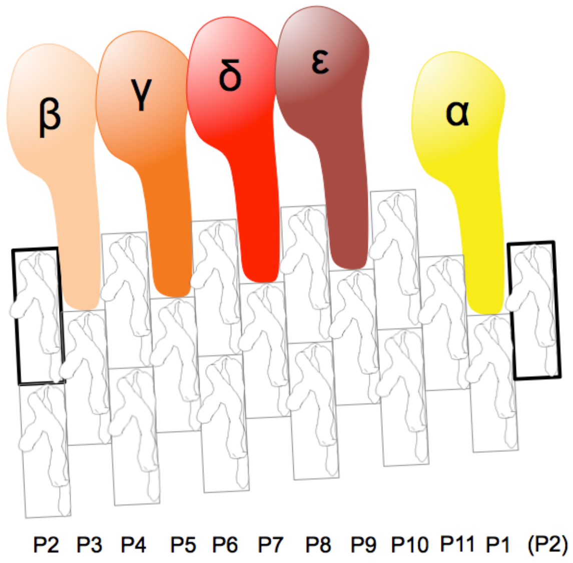

Figure S7. Protofilament and IpaD assignments in this study. (A) 2D representation of the needle filament, with protofilaments labelled from P1-P11, with P1 corresponding to the lowest protofilament in the helical arrangement. Subunits boxed in bold line are at an identical location in the needle helix. IpaD subunits are labeled from α → ε according to the proposed order of insertion (with α being the first subunit inserted). IpaDs are colour-coded based on the heights of the subunits, with yellow indicating the subunit bound to the lowest binding position (P1) and maroon indicating the subunit bound to the highest binding position (P9). **Validation of 3D maps by matching of projections with class averages.** The WT 3D **(B)** and Δ*ipaB* **(C)** maps were projected every 72°, with each projection corresponding to one subunit. The class averages that were used for the reconstructions were then searched to find the class averages that correspond to each view of the map projections.

B

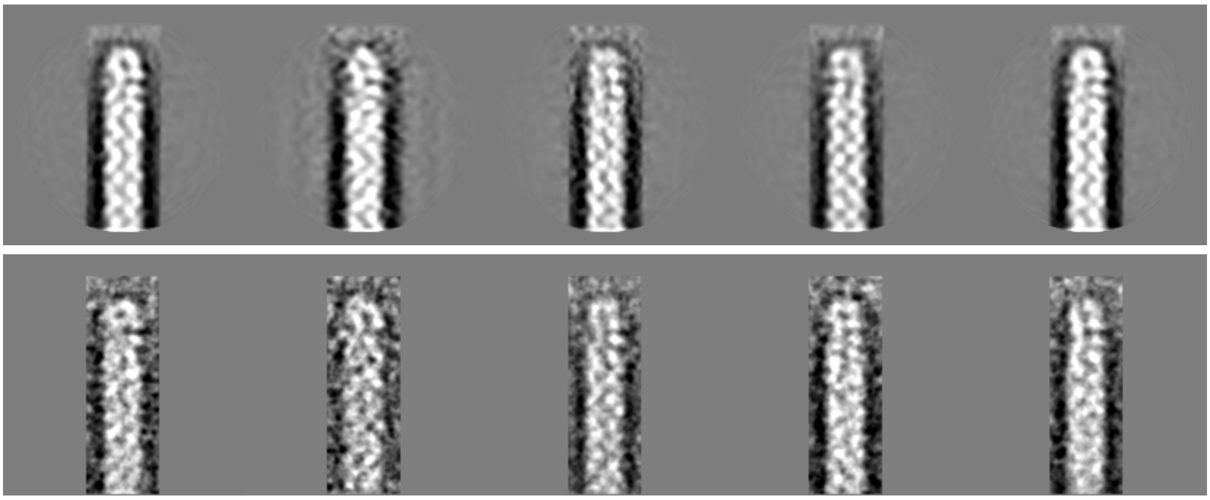

C

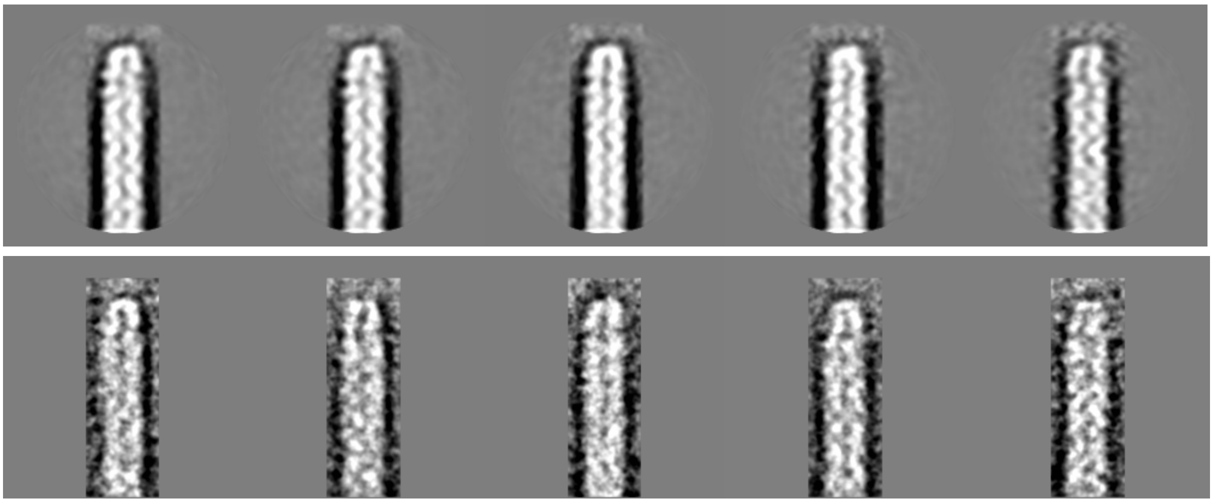

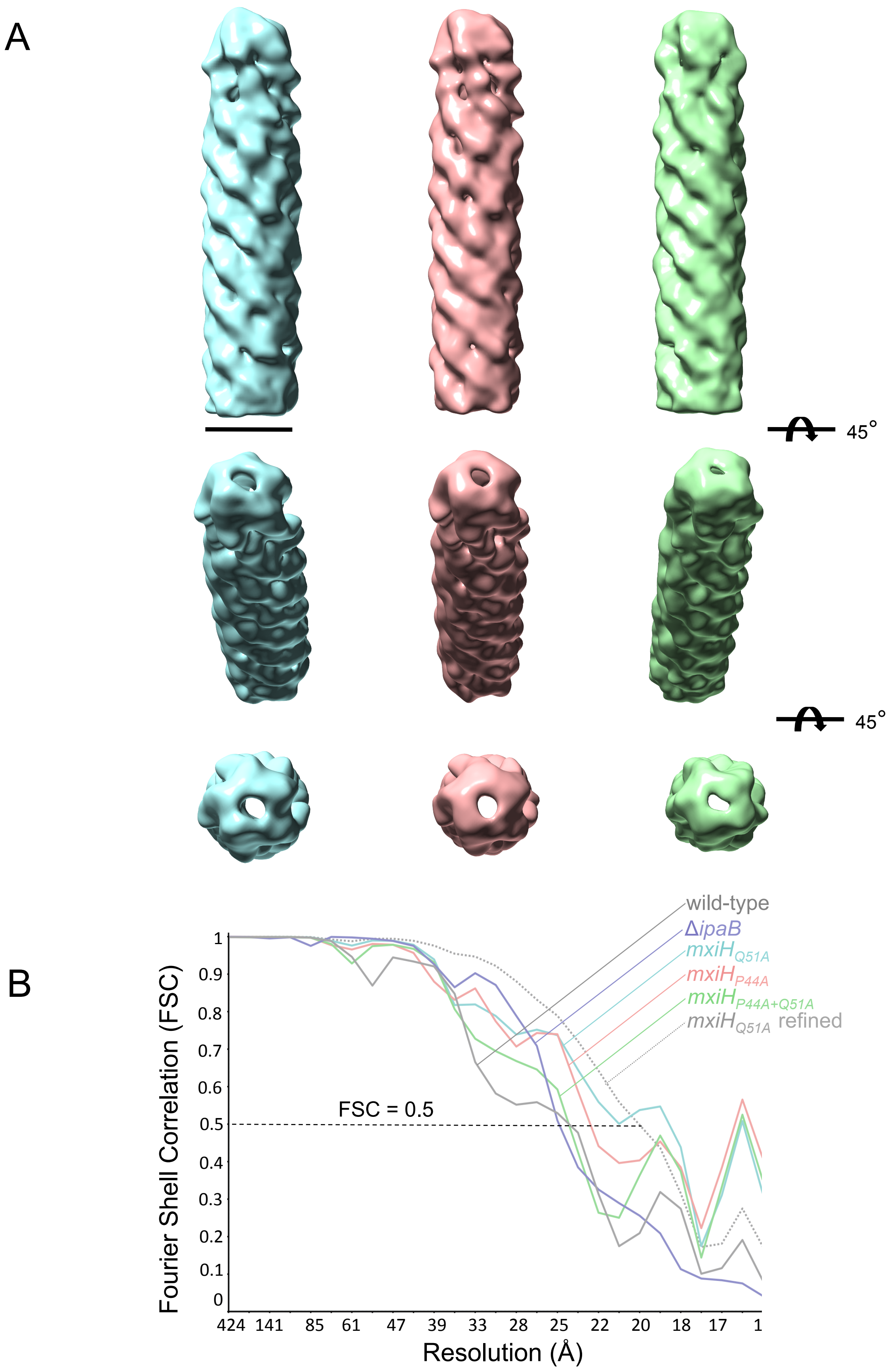

Figure S8. 3D maps of MxiH mutant TCs. (A) Side and rotated views of *mxiH*$_{Q51A}$ (blue), *mxiH*$_{P44A}$ (pink) and *mxiH*$_{P44A+Q51A}$ (green). Maps displayed at a contour levels of 0.0332, 0.0354 and 0.0306 in Chimera, respectively. **(B)** Estimation of map resolutions by Fourier Shell Correlation (FSC). Scale bar, 70 Å.

A

```
IpaD3           MNITTLTNSISTSSFSPNNTNGSSTETVNSDIKTTTSSHPVSSLTMLNDTLHNIRTTNQA 60
IpaD6           MNITTLTNSISTSSFSPNNTNGSSTETVNSDIKTTTSSHPVSSLTMLNDTLHNIRTTNQA 60
IpaD1           MNITTLTNSISTSSFSPNNTNGSSTETVNSDIKTTTSSHPVSSLTMLNDTLHNIRTTNQA 60
IpaD2           MNITTLTNSISTSSFSPNNTNGSSTETVNSDIKTTTSSHPVSSLTMLNDTLHNIRTTNQA 60
IpaD7           MNITTLTNSISTSSFSPNNTNGSSTETVNSDIKTTTSSHPVSSLTMLNDTLHNIRTTNQA 60
IpaD8           MNITTLTNSISTSSFSPNNTNGSSTETVNSDIKTTTSSHPVSSLTMLNDTLHNIRTTNQA 60
IpaD5           MNITTLTNSISTSSFSPNNTNGSSTETVNSDIKTTTSSHPVSSLTMLNDTLHNIRTTNQA 60
IpaD4           MNITTLTNSISTSSFSPNNTNGSSTETVNSDIKTTTSSHPVSSLTMLNDTLHNIRTTNQA 60
                ************************************************************

IpaD3           LKKELSQKTLTKTSLEEIALHSSQISMDVNKSAQLLDILSRNEYPINKDARELLHSAPKE 120
IpaD6           LKKELSQKTLTKTSLEEIALHSSQISMDVNKSAQLLDILSRNEYPINKDARELLHSAPKE 120
IpaD1           LKKELSQKTLTKTSLEEIALHSSQISMDVNKSAQLLDILSRHEYPINKDARELLHSAPKE 120
IpaD2           LKKELSQKTLTKTSLEEIALHSSQISMDVNKSAQLLDILSRNEYPINKDARELLHSAPKE 120
IpaD7           LKKDLSQKTLTKTSLEEIALHSSQISMDVNKSAQLLDILSKKEYPINKDARELLHSAPKE 120
IpaD8           LKKDLSQKTLTKTSLEEIALHSSQISMDVNKSAQLLDILSKKEYPINKDARELLHSAPKE 120
IpaD5           LKKDLSQKTLTKTSLEEIALHSSQISMDVNKSAQLLDILSKKEYPINKDARELLHSAPKE 120
IpaD4           LKKDLSQKTLTKTSLEEIALHSSQISMDVNKSAQLLDILSKKEYPINKDARELLHSAPEE 120
                ***:************************************::****************:*

IpaD3           AELDGDQMISHRELWAKIANSINDINEQYLKVYEHAVSSYTQMYQDFSAVLSSLAGWISP 180
IpaD6           AELDGDQMISHRELWAKIANSINDINEQYLKVYEHAVSSYTQMYQDFSAVLSSLAGWISP 180
IpaD1           AELDGDQMISHRELWAKIANSINDINEQYLKVYEHAVSSYTQMYQDFSAVLSSLAGWISP 180
IpaD2           AELDGDQMISHRELWAKIANSINDINEQYLKVYEHAVSSYTQMYQDFSAVLSSLAGWISP 180
IpaD7           AELDGYEMISHRELWDKIAKSINNINEQYLKVYEHAVSSYTQMYQDFSAVLSSLAGWISP 180
IpaD8           AELDGYEMISHRELWDKIAKSINNINEQYLKVYEHAVSSYTQMYQDFSAVLSSLAGWISP 180
IpaD5           AELDGYEMISHRELWDKIAKSINNINEQYLKVYEHAVSSYTQMYQDFSAVLSSLAGWISP 180
IpaD4           AELDGYQMISHRELWDKIAKSINNINEQYLKVYEHAVSSYTQMYQDFSAVLSSLAGWISP 180
                ***** :******** ***:***:************************************

IpaD3           GGNDGNSVKLQVNSLKKALEELKEKYKDKPLYPANNTVSQEQANKWLTELGGTIGKVSQK 240
IpaD6           GGNDGNSVKLQVNSLKKALEELKEKYKDKPLYPANNTVSQEQANKWLTELGGTIGKVSQK 240
IpaD1           GGNDGNSVKLQVNSLKKALEELKEKYKDKPLYPANNTVSQEQANKWLTELGGTIGKVSQK 240
IpaD2           GGNDGNSVKLQVNSLKKALEELKKKYEDKPLYPATNTVSQKEADKWLTELGGTIGKVSKK 240
IpaD7           GGNDGNSVKLQVKSLKDELTKLKEKYKDKPLYPANNTVSKEQANKWLTELGGTIGKVSEK 240
IpaD8           GGNDGNSVKLQVKSLKDELTKLKEKYKDKPLYPANNTVSKEQANKWLTELGGTIGKVSEK 240
IpaD5           GGNDGNSVKLQVKSLKDELTKLKEKYKDKPLYPANNTVSKEQANKWLTELGGTIGEVSEK 240
IpaD4           GGNDGNSVKLQVNSLKAELTKLKEKYEDKPLYPANNTVSKEQADKWLTELGGTIGTVSRK 240
                ************:***  * :**:**:*******.****:::*:*********** **.*

IpaD3           NGGYVVSINMTPIDNMLKSLDNLGGNGEVVLDNAKYQAWNAGFSAEDETMKNNLQTLVQK 300
IpaD6           NGGYVVSINMTPIDNMLKSLDNLGGNGEVVLDNAKYQAWNAGFSAEDETMKNNLQTLVQK 300
IpaD1           NGGYVVSINMTPIDNMLKSLDNLGGNGEVVLDNAKYQAWNAGFSAEDETMKNNLQTLVQK 300
IpaD2           NGGYVVNINMTPIDNMLKSLNNLGGNGEVVLDNAKYQAWNAGFSAEDETMKNNLQTLVQK 300
IpaD7           NGGYVVNINMTPIDNTLKSLDNLGGNGEVVLDNAKYQAWNAGFSAEDETMKNNLQTLVQK 300
IpaD8           NGGYVVNINMTPIDNMLKSLDNLGGNGEVVLDNAKYQAWNAGFSAEDETMKNNLQTLVQK 300
IpaD5           NGGYVVNINMTPIDNMLKSLDNLGGNGEVVLDNAKYQAWNAGFSAEDETMKNNLQTLVQK 300
IpaD4           NGGYVVNINMSPIDNMLKSLNNLGGNGEVVLDNAKYQAWNAGFSAEDETMKNNLQTLVQK 300
                ******.***:**** ****:***************************************

IpaD3           YSNANSIFDNLVKVLSSTISSCTDTDKLFLHF 332
IpaD6           YSNANSIFDNLVKVLSSTISSCTDTDKLFLHF 332
IpaD1           YSNANSIFDNLVKVLSSTISSCTDTDKLFLHF 332
IpaD2           YSNANSIFDNLVKVLSSTISSCTDTDKLFLHF 332
IpaD7           YSNANSIFDNLVKVLSSTISSCTDTDKLFLHF 332
IpaD8           YSNANSIFDNLVKVLSSTISSCTDTDKLFLHF 332
IpaD5           YSNANSIFDNLVKVLSSTISSCTDTDKLFLHF 332
IpaD4           YSNANSIFDNLVKVLSSTISSCTDTDKLFLHF 332
                ********************************
```

B

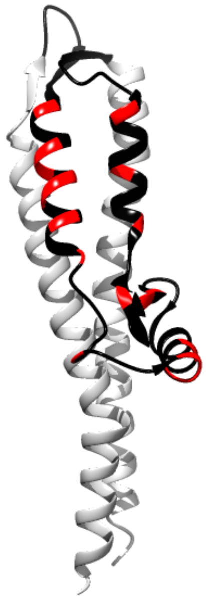

C

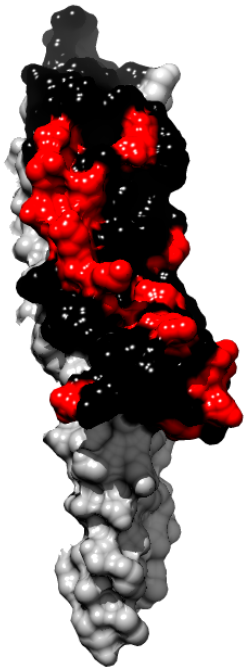

Figure S9. Sequence variation within IpaD. A) Alignment of all available IpaD sequences. This was done using ClustalW2 from the EBI Server. IpaD1 is from *Shigella flexneri* 2a str. 301, D2 from *S. sonnei* Ss046, D3 from pWR501, D4 from *S. dysenteriae* Ss197, D5 from *S. boydii* CDC 3083-94, D6 from *S. flexneri* pWR100, D7 from *Shigella* serotype 4 and D8 from *Shigella* seroptype 6. The only regions in IpaD showing any variability are between aa 125 and 135 (which corresponds to a small flexible loop region) and between aa 190 and 260. The latter region corresponds to helices 4 to 6 (inclusive) in the protein structure or the entire globular C-terminal domain, which is also the region recognised by anti-LcrV and PcrV antibodies leading to protection against *Yersinia* and *Pseudomonas*. B) Ribbon and C) surface representation of IpaD crystal structure (PDB 2j0n, chain B; Johnson et al., 2007). The globular C-terminal domain is shown in black, the variable amino acids identified in A are in red and the remainder of the protein is in light gray. All but one variable residue are surface exposed. The almost entirely red helix is helix α4.
